# Exosomal STIMATE derived from type II alveolar epithelial cells controls metabolic reprogramming of tissue-resident alveolar macrophages

**DOI:** 10.7150/thno.82552

**Published:** 2023-01-22

**Authors:** Zunyong Feng, Zhou Jing, Qiang Li, Liuxi Chu, YuXin Jiang, Xuanbo Zhang, Liang Yan, Yinhua Liu, Jing Jiang, Ping Xu, Qun Chen, Ming Wang, Hui Yang, Guoren Zhou, Xiaochun Jiang, Xiaoyuan Chen, Hongping Xia

**Affiliations:** 1 School of Biological Sciences and Medical Engineering & Zhongda Hospital, School of Medicine, Advanced Institute for Life and Health & Interdisciplinary Innovation Institute for Medicine and Engineering, Southeast University, Nanjing, China.; 2 The Translational Research Institute for Neurological Disorders & Interdisciplinary Research Center of Neuromedicine and Chemical Biology of Wannan Medical College and Anhui Normal University, Department of Neurosurgery, the First Affiliated Hospital of Wannan Medical College (Yijishan Hospital of Wannan Medical College), Wuhu, China.; 3 Departments of Diagnostic Radiology, Surgery, Chemical and Biomolecular Engineering, and Biomedical Engineering, Clinical Imaging Research Centre, Nanomedicine Translational Research Program, Yong Loo Lin School of Medicine and College of Design and Engineering, National University of Singapore, Singapore, Singapore. Institute of Molecular and Cell Biology, Agency for Science, Technology, and Research (A*STAR), Singapore, Singapore.; 4 Department of Pathology, School of Basic Medical Sciences & Sir Run Run Hospital & Key Laboratory of Antibody Technique of National Health Commission, Nanjing Medical University, Nanjing, China.; 5 Department of Pathology & Central Laboratory Intensive & Care Unit, The First Affiliated Yijishan Hospital of Wannan Medical College, Wuhu, China.; 6 Department of Anatomy & Biochemistry and Molecular Biology, Wannan Medical College, Wuhu, China.; 7 Department of Pathogenic Biology and Immunology, School of Medicine, Jiaxing University, Jiaxing, China.; 8 Department of Neurosurgery, The Second Xiangya Hospital, Central South University, Changsha, China.; 9 Department of Oncology, Jiangsu Cancer Hospital and The Affiliated Cancer Hospital of Nanjing Medical University and Jiangsu Institute of Cancer Research, Nanjing, China.

**Keywords:** STIMATE, Exosome, ALI/ARDS, Alveolar macrophages, Type 2 alveolar epithelial.

## Abstract

**Background:** Complete abolition of alveolar epithelial cells (AECs) is characteristic of end-stage lung disease. Transplantation therapy of type II AECs (AEC-IIs) or AEC-IIs-derived exosomes (ADEs) have been proposed as a means of repairing injury and preventing fibrosis. However, the mechanism by which ADEs balances airway immunity and alleviates damage and fibrosis remains unknown.

**Methods:** We investigated STIM-activating enhancer-positive ADEs (STIMATE^+^ ADEs) in the lung of 112 ALI/ARDS and 44 IPF patients, and observed the correlation between STIMATE^+^ ADEs and subpopulation proportion and metabolic status of tissue-resident alveolar macrophages (TRAMs). We constructed the conditional knockout mice STIMATE*^sftpc^*, in which STIMATE was specifically knocked out in mouse AEC-IIs and observed the effects of STIMATE^+^ ADEs deficiency on disease progression, immune selection and metabolic switching of TRAMs. We constructed a BLM-induced AEC-IIs injury model to observe the salvage treatment of damage/fibrosis progression with STIMATE^+^ ADEs supplementation.

**Results:** In clinical analysis, the distinct metabolic phenotypes of AMs in ALI/ARFS and IPF were significantly perturbed by STIMATE^+^ ADEs. The immune and metabolic status of TRAMs in the lungs of STIMATE*^sftpc^* mice was imbalanced, resulting in spontaneous inflammatory injury and respiratory disorders. STIMATE^+^ ADEs are taken up by tissue-resident alveolar macrophages TRAMs to regulate high Ca^2+^ responsiveness and long-term Ca^2+^ signal transduction, which maintains M2-like immunophenotype and metabolism selection. This involves calcineurin (CaN)-PGC-1α pathway mediated mitochondrial biogenesis and mtDNA coding. In a bleomycin-induced mouse fibrosis model, supplementation with inhaled STIMATE^+^ ADEs lessened early acute injury, prevented advanced fibrosis, alleviated ventilatory impairment and reduced mortality.

## Introduction

Alveolar epithelial cells (AECs) are barriers that isolate the body fluid environment from the external environment and are often characterized by signs of danger, such as microorganisms, damaged organelles or drugs. A total of 95% of AECs are thin-walled type I alveolar epithelial cells (AEC-Is) and rectangular type II alveolar epithelial cells (AEC-IIs) 1-2. AEC-IIs are local unipotent stem cells that differentiate into pulmonary AEC-Is after cell barrier injury 3 and maintain lung tone and nonprofessional phagocytosis in innate immunity and immune homeostasis. Failed AEC-IIs vitality results in unusual molecular endophenotypes and cellular signatures 4, such as perpetuation of inflammatory cycles and poor resolution of the damaged tissue environment, which manifests itself in two forms of clinical diseases 5-6, namely, acute lung injury/acute respiratory distress syndrome (ALI/ARDS) and interstitial pulmonary fibrosis (IPF).

ALI/ARDS remains the greatest clinical challenge in intensive care units 7-8. Mechanical ventilation is helpful, but the death rate remains high and there is no effective interventional drug 9. Most ARDS patients survive the acute phase but still die from the fibrosis that ensues 10. Progressive pulmonary fibrosis also contributes to the poor quality of life of ARDS survivors 11-12. Prophylactic intervention for impending pulmonary fibrosis during the acute injury phase is beneficial for improving long-term survival and prognosis in ALI/ARDS. The progenitor properties of AEC-IIs suggest that they have great potential in the treatment of airway diseases. Transplantation of AEC-IIs into ALI/ARDS or lung fibrosis mice abrogated lung injury, demonstrated by blood oxygen recovery, decreased collagen deposition, and increased survival 13-14. However, the availability of donor AEC-IIs for transplantation is often limited by cost, donor scarcity and rejection reactions.

Resolution of lung inflammation and remodeling of the tissue fibrosis environment requires cooperation between different cell types, among which alveolar macrophages (AMs) play a central role. Inflammatory AMs (also known as M1 AMs) are engaged in the initial response to injury and infection, and alternatively activated AMs (M2 AMs) are essential for wound closure and the resolution of tissue repair. The crosstalk between their metabolic state and metabolic reprogramming determines the fate of stromal cells and regulates their role in lung injury repair 15. Tissue-resident AMs (TRAMs) are derived from the yolk sac and fetal liver cells. As long-lived cells, they can proliferate *in situ* to maintain their own stability 16-17. TRAMs continuously switch their immune and metabolic states to challenge the complex and harsh alveolar environment, thus avoiding unnecessary inflammatory responses and tissue fibrosis 18-19. The local turnover rate of TRAMs in adult lungs is 40% per year 20, which is especially important for elderly individuals. Impaired TRAMs are the direct cause of most chronic airway diseases 21.

Crosstalk between AMs and AECs is essential to maintain lung homeostasis 22. Exosomes have attracted much attention as signal mediators between AMs and AECs. For example, AECs can take up SOCS1/3 contained in exosomes secreted from AMs to control the inflammatory response of lung disorders 23. AMs secrete and transmit exosomal leukotriene from AECs, induce proliferation, chemoattract inactivated macrophages, and aggravate asthma-type lung injury 24. Stem cell-derived exosomes have demonstrated active therapeutic potential in tissue repair, inflammation regulation, and proliferation inhibition 25-27, especially in the cardiopulmonary circulation. Recent studies have shown that bronchial epithelial cell-derived exosomes ameliorate pulmonary fibrosis and ALI in mice. Our previous study also showed that signaling crosstalk between AEC-IIs-derived exosomes and AMs maintains airway immune balance 28. However, additional studies to observe the potential effects of AEC-IIs-derived exosomes in the treatment of airway diseases characterized by lung injury-pulmonary fibrosis have not been conducted.

In this study, we observed crosstalk between AEC-IIs and TRAMs mediated by STIMATE-containing exosomes, and found their regulatory role in AM metabolic reprogramming and immune homeostasis in IPF and ALI/ARDS. Considering the low cost, low immunogenicity and controllable production protocol of exosomes, STIMATE-positive AEC-IIs-derived exosomes (STIMATE+ ADEs) can be used as an alternative for stem cell transplantation.

## Materials and methods

### ALI/ARDS and IPF cohorts

BALF samples of ALI/ARDS and PF patients were obtained from the Intensive Care Unit of Yijishan Hospital Affiliated to Wannan Medical College, ALI/ARDS were screened according to the Berlin definition standards 29, and IPF patients were screened according to the American Thoracic Society (ATS) and the European the Respiratory Society (ERS) 30. The baseline clinical characteristics of all patients are listed in Supplementary Table 1 and Supplementary Table 2. All experimental protocols using human specimens were approved by the Medical Ethics Review Committee of Wannan Medical College. Informed consent was obtained from all subjects. The research complies with the principles outlined in the Declaration of Helsinki. Detailed processing of human samples and assays for biochemical indicators are presented in the Supplementary Materials.

### Transgenic mice and induced lung injury model

CRISPR/Cas-mediated genome engineering was used to establish conditional STIMATE (NCBI reference sequence: NM_028839.4; Ensembl: ENSMUSG00000006526)-null allele mice (C57BL/6J background), deleting the exon 2 and 3 regions in Cre-mediated recombination. The deletion of STIMATE exons 2 and 3 will cause the loss of function of the STIMATE gene, and the cKO region sequence does not have any other known genes. Cas9, gRNA, and targeting vector will be injected into the fertilized egg to produce cKO mice. The pups will be genotyped by PCR and then sequenced and analyzed. Two STIMATE^flox/+^ chimeras were crossed to obtain STIMATE^flox/flox^ homozygous mice. To obtain conditional STIMATE-deficient AEC-IIs cells, STIMATE^flox/flox^ mice were crossed with SFTPC-cre tool mice (AEC-IIs cell-specific expression) to produce STIMATE^flox/+^; SFTPC-cre mice were further backcrossed to produce STIMATE^flox/flox^; SFTPC-cre (STIMATE*^sftpc^*) homozygous mice. Due to the lack of immune function of STIMATE*^sftpc^*, it is strictly kept in an SPF animal room. SFTPC-cre tool mice were used as described previously 28. Details of the experimental animal design are provided in the Supplementary Materials. All protocols involving animals in this study were approved by the Institutional Animal Care and Use Committee (IACUC) of Wannan Medical College and were carried out in accordance with the approved guidelines.

### Cultivation of primary AEC-IIs and TRAMs

Primary sorting and cultivation of AEC-IIs to obtain STIMATE^+^ ADE 31. The single-cell suspension preparation procedure is presented in the Supplementary Materials. A FACSVerse flow cytometer (BD Biosciences, NJ, USA) was used to sort the live single-cell suspension to obtain high-purity AT II (Lin- MHCII^+^EpCAM^+^) ^58^. The sorted AEC-IIs were cultured in keratinocyte-SFM medium (Gibco, CA, USA) containing 1 mM isoproterenol, 10 μg/ml fibronectin, 30 μg/ml vitamins and 10 μg/ml BSA. For the primary culture of TRAMs, the preparation of a single-cell suspension is shown above, and the sorting strategy is reported 36. The antibody information required for sorting is shown in Supplementary Table S3. The sorted TRAMs and BMDMs were cultured in 10% heat-inactivated fetal bovine serum, 2 mM L-glutamine, 1 mM pyruvate, 50 μM 2-mercaptoethanol, 100 U/ml penicillin, and 100 μg/ml streptomyces RPMI 1640 medium (HyClone, UT, USA). All cells were cultured in a constant temperature incubator with 5% CO2 and 37 °C. For the culture of MLE-12 cells, MLE-12 was obtained from ATCC (CA, USA) and cultured in HITES medium supplemented with 2% FBS.

### Gene knockout, coexpression and luciferase plasmid vector construction and transfection

The CRISPR-Cas9 genome editing tool was used to knock out STIMATE and Rab27a under the guidance of sgRNA and synthesize sgRNA targeting mouse STIMATE exon 4 and mouse Rab27a exon (GenePharma, Shanghai, CHA) in TRAMs and AEC-II, respectively. STIMATE-GFP was generated through gateway cloning by using the vectors pCDNA-DEST54 and STIMATEp.K241Q mutant constructs were subsequently made by using the QuikChange Lightning Multi site-directed mutagenesis kit (Agilent). The luciferase reporter gene pRL-TK-PGC-1α-promoter plasmids were purchased from Promega (WI, USA). pGH1-PGC-1 shRNA and the corresponding shRNA NC interference plasmid were synthesized with the assistance of GenePharma (Shanghai, CHINA). One microgram of each plasmid was transfected into AEC-IIs or TRAMs using Lipofectamine 3000 (Merck, MA, USA). After 48 hours, the transfection efficiency was detected by fluorescent labeling, and gene disruption was detected by Western blotting or flow cytometry. The sgRNA sequence and shRNA sequence can be found in Supplementary Table S4.

### BALF exosome extraction, enrichment, identification, quantification, proteomics analysis

Ultrafiltration centrifugation was used to obtain exosomes in BALF 32. The separated exosomes were resuspended in 100 μL of PBS (per 20 ml sample solution) for subsequent experiments. Western blotting was used to identify the surface markers of cell exosomes (CD63 and TSG101) and to detect endoplasmic reticulum and lysosomal pollutant markers (Erp72 and Lamp1). The antibody product information is shown in Supplementary Table S3. Nanoparticle tracking analysis (NTA) NanoSight NS300 (Malvern Instruments, England) was used to obtain the particle size and quantity of exosomes purified from BALF or cell culture supernatant. The purified exosomes were placed on a nickel grid coated with formvar carbon and stained with 2% uranyl acetate. The grid was air-dried and observed using a JEM-1230 transmission electron microscope (TEM) (JEOL, Tokyo, Japan). Exosomal proteomics was performed by Aptbiotech (Shanghai, China). Grouping data and differentially expressed protein data can be obtained in Supplementary Table S5. Gene ontology analysis was performed using the online website DAVID (https://david.ncifcrf.gov/). More detailed extracellular vesicle processing procedures can be found in the Supplementary Materials.

### Analysis of Cell Metabolites

Metabolite analysis of TRAMs was performed on a Thermo Fisher Scientific Triple Quadrupole LC-MS (Thermo Fisher Scientific, MA, USA) system. In short, 24 h before LC-MS, WT or STIMATE-/- TRAMs were seeded on a 6-well plate at a medium density. After preincubating TRAMs with STIMATE+-ADEs of different genetic backgrounds for 12 h, 100 ng/ml rm GM-CSF was added to induce proliferation for 12 h. The TRAM cell pellet was centrifuged at 1200 × g for 5 min and washed with precooled PBS. One milliliter of ice-cold 80% methanol containing 10 ng/ml internal standard valine-d8 was used to extract cellular metabolites. The precipitate was collected after centrifugation at 10000 × g for 10 min, dried in a benchtop vacuum centrifuge (Eppendorf, Hamburg, Germany) and dissolved in 100 ml ultrapure water. One milliliter of the sample was injected into a ZIC-pHILIC 2.1×150 mm chromatographic column (Millipore, MA, USA). The buffer was 20 mM ammonium carbonate + 0.1% ammonium hydroxide (Buffer A) and acetonitrile (Buffer B). The chromatographic gradient was performed at a flow rate of 0.0357 ml/min, and 20% Buffer A was increased to 80% in a time gradient from 0-20 min. At 20-20.5 min, 80% Buffer A reached 20% in a time gradient. At 20.5-28 min, hold 20% of Buffer A. The mass spectrometer operates in full scan, polarity switching mode. XCalibur QuanBrowser 2.2 (Thermo Fisher Scientific, MA, USA) was used to refer to the internal chemical standard library for relative quantification of polar metabolites. The relative abundance of metabolites in the cell was obtained by normalizing the analysis results.

### Detection of mitochondrial copy number

A FlexiGene DNA kit (Qiagen, Duesseldorf, GER) was used to isolate total DNA from cells. Oligonucleotide probes were designed for 3 different regions of mitochondrial DNA (mtDNA) and 2 regions of genomic DNA. The specific primer sequence is shown in Supplementary Table S4. Maxima SYBR Green qPCR Master Mix (Thermo Fisher Scientific, Waltham, MA, USA) was used for real-time quantitative PCR. The copy number of mtDNA was calculated relative to the amount of genomic DNA according to the method of Maus et al. 33.

### Endolysosomal TLR7/9 cleavage induced by mtDNA/rmSP-A agarose beads

Streptomycin affinity agarose-coated mtDNA beads were provided by Polysciences, biotin-labeled recombinant mouse SP-A was purchased from MedChemExpress, and biotin-labeled mCherry and GFP were purchased from Merck. MLE-12 (1x10^7^) was incubated with ~10^7^ 1 μM latex beads (Polysciences, MA, USA) at various time points. Fluorescence images of mRFP and GFP were captured directly using an inverted fluorescence microscope. For detection of TLR7/TLR9 cleavage activation in endolysosomes, after incubating mtDNA/rmSP-A agarose beads for the indicated times, PBS washes were scraped into 3 ml of sucrose homogenization buffer and pelleted by centrifugation. Resuspend the cells in 1 ml of SHB plus protease inhibitor cocktail (Roche) and EDTA and disrupt the cells in a steel Duane homogenizer. Intact cells, nuclei, and total debris were then removed by centrifugation at 8,000 × g. The remaining supernatant was further processed to isolate intact phagolysosomes. Fifty microliters were set aside as a "lysate" sample for SDS electrophoresis and western blot analysis. The remaining supernatant was mixed with an equal volume of 60% sucrose, and a sucrose gradient was applied as follows: 1 ml 60%, 2 ml 20%, 2 ml 32%, 2 ml 20%, and 2 ml 10%. Phagolysosomes were harvested from the 20%-10% sucrose interface after the gradient was spun at 100,000 × g for 1 h. Protein concentrations were normalized by BCA assay, followed by SDS electrophoresis and Western blot analysis.

### Custom Sandwich-based Antibody Arrays

Custom sandwich-based antibody arrays were provided by Raybiotech (GA, USA) and implemented in accordance with the instructions. In short, each membrane of the antibody array was placed into the well of the culture tray, 2 ml of blocking buffer was transferred into each well, and the plate was incubated at room temperature for 30 min. One milliliter of TRAM cell sample was pipetted from the cell lysate (Sigma-Aldrich, MO, USA) into each well and incubated at 4 °C overnight. After removing the sample, the washing was repeated 3 times with 20x washing buffer. Transfer 1 ml of biotin-labeled antibody mixture into each well and incubate overnight at 4 °C. After removing the biotin-labeled antibody, the cells were washed 3 times. Two milliliters of 1,000× HRP-streptavidin were added to each well and incubated at room temperature for 2 h. This process was repeated 3 times after washing. Pipette 500 µl of the detection buffer mixture onto each membrane and incubate for 2 min at room temperature. The sandwich film was transferred to a chemiluminescence imaging system with a CCD camera for exposure. ImageJ software (National Institutes of Health, MD, USA) was used to analyze the gray value of each film hole. All incubations and washes were performed on a shaker at 0.5-1 cycle/sec. The antibody target of each well is shown in FIGURE S5B.

### RNA isolation and polysome experiments

A polysome analysis experiment 35 was used to detect the efficiency of mRNA translation into proteins. In short, AEC-IIs were pretreated with 100 μg/ml cycloheximide (Sigma-Aldrich, MO, USA) for 10 min. Lysis buffer (10 mM Tris-HCl pH 8, 140 mM NaCl, 1.5 mM MgCl2, 0.25% NP-40, 0.1% Triton X-100, 50 mM DTT, 150 μg/ml cyclohexanamide and 640 U/ml RNAsin) was used to treat the cells for 30 min and centrifuged at 10,000 g to obtain the supernatant. The supernatant was transferred to a 10-50% sucrose filler gradient centrifuge tube. After centrifugation at 35,000 × g for 3 h, qPCR (Applied Biosystems, CA, USA) was used to detect the mRNA level of STIMATE in each sucrose layer.

### Statistics and analysis

All numerical results are the mean ± standard deviation (SD). The statistical significance of the differences between the experimental groups was determined by the student's t-test or analysis of variance (ANOVA) followed by a post hoc test or a special test method, such as the respective figure legend. Differences between p value <0.05 (marked as * in the figure) and p <0.01 (**) are considered significant. The number of replicate experiments and mice in each group is shown in the respective legend. For more details and other methods, see supplementary information.

## Results

### Characterization and clinical significance of STIMATE+ exosome released by AEC-IIs

**Figure 1:**
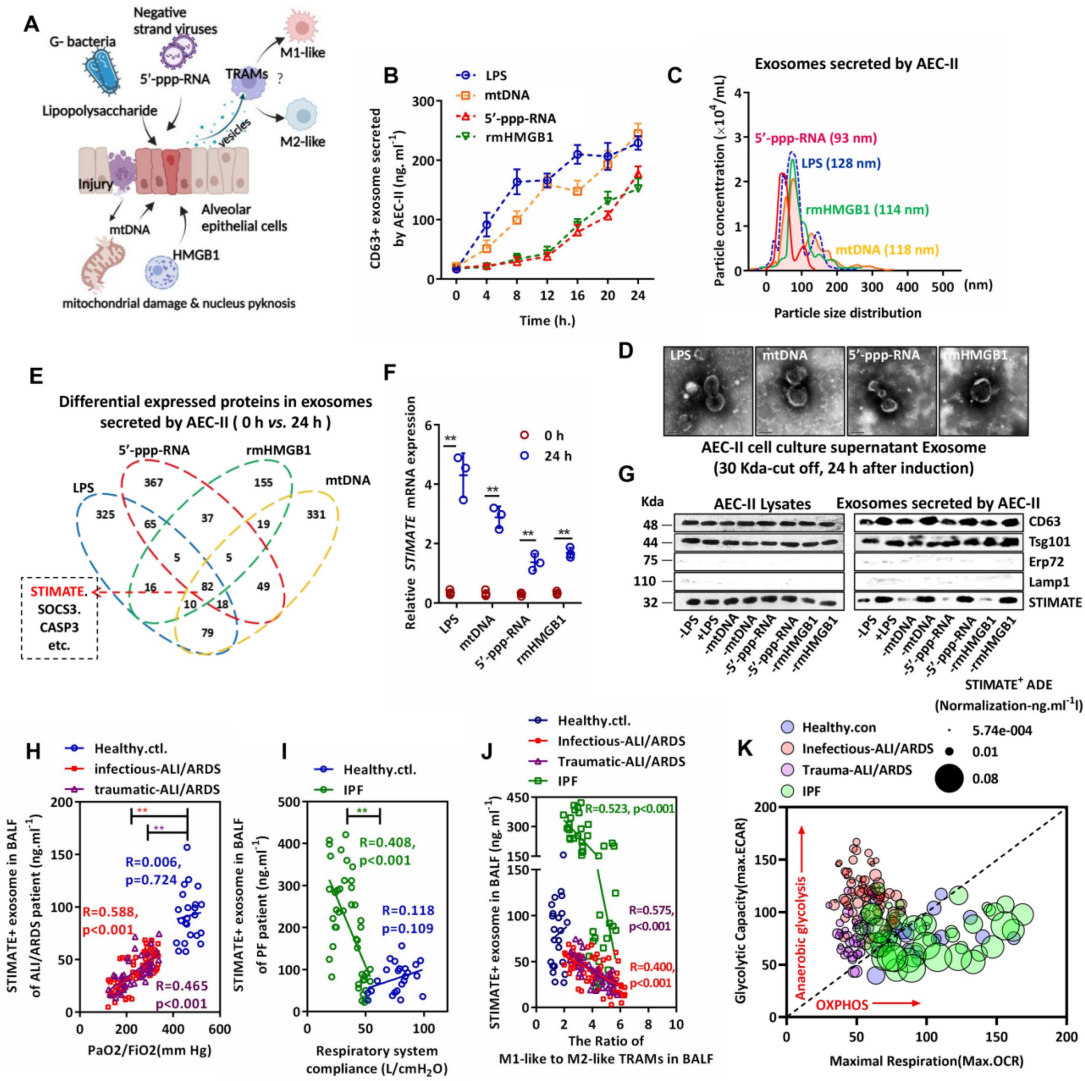
*** Characterization and clinical monitoring of STIMATE^+^ exosomes released by human AEC-IIs. (A)***Schematic diagram of mouse AEC-II perturbed by endogenous or exogenous risk factors and crosstalk with TRAMs through exosome secretion. **(B)**The time-dependent quantitative polyline of CD63^+^ exosomes released by mouse primary mouse AEC-IIs stimulated by LPS & rmHMGB1 and transfected mtDNA & 5'-pppRNA (n = 6), drawn according to the standard curve of recombinant CD63. **(C)**A NanoSight nanoparticle tracking system characterizes the release of ultrafiltration centrifugation of exosomes from mouse AEC-IIs stimulated by LPS & rmHMGB1 and mtDNA & 5'-pppRNA.** (D)**Representative TEM image of mouse AEC-IIs-releasing exosomes purified by ultrafiltration centrifugation.** (E)**Venn diagram of the overlap of differential protein cargo in mouse AEC-IIs release of exosomes by proteomics analysis stimulated by LPS & rmHMGB1 and transfected by mtDNA & 5'-pppRNA.** (F)**STIMATE mRNA in mouse AEC-II cells detected by qPCR in the presence or absence of LPS & rmHMGB1 and mtDNA & 5'-pppRNA (n = 3). **(G)**Representative Western blotting image of STIMATE protein expression in mouse AEC-IIs and exosomes (n = 3). CD63 and Tsg101 represent Exo-specific markers, and ERP27 and Lamp1 represent ER and lysosomal contamination markers, respectively. **(H)**STIMATE-positive AEC-IIs-derived exosomes (STIMATE+ ADEs) in lung BALF from healthy subjects (n = 23), infectious ALI/ARDS patients (n = 72) and traumatic ALI/ARDS patients (n = 40) were detected by exosome-based ELISA. *The abscissa is the* PaO_2_/FiO_2_* (representing the ventilation capacity) when the BALF sample was collected from each patient.* Linear correlation between STIMATE*^+^* Exo concentration and PaO_2_/FiO_2_ was analyzed by Pearson's correlation. ***(I)***STIMATE^+^ ADEs in lung BALF from healthy subjects (n = 23) and interstitial PF patients (n = 44). *The abscissa* axis *is the ventilator records of* respiratory system compliance (Rsc,* represents lung tension*)* of each patient.* Linear correlation between STIMATE*^+^* Exo concentration and Rsc was analyzed by Pearson's correlation. ***(J)*** The ratios of M1-like to M2-like TRAMs in lung BALF from healthy subjects (n = 23), infectious ALI/ARDS patients (n = 72), traumatic ALI/ARDS patients (n = 40) and interstitial PF patients (n = 44). ***(K)***Maximal OCR plotted against maximal ECAR from the data in (Supplementary materials Figure S3C and S3D), and the bubble size indicates the relative amount of STIMATE^+^ ADEs*.*Data are shown as the mean ± SD, *p < 0.05 and **p < 0.01 by Student's t test (f), analysis of variance (ANOVA) followed by a post hoc test (i) or Pearson correlation analysis (j, k, l). All cell experiment data were derived from 3 independent experiments.

As shown in FIGURE 1A, to simulate the response of AEC-IIs to inflammatory injury danger signs in the alveolar cavity, we used representative endogenous damage-associated molecular patterns (DAMPs) [mitochondrial DNA (mtDNA) and nuclear rmHMGB1] and exogenous pathogen-associated molecular patterns (PAMPs) [gram-negative bacterial lipopolysaccharide (LPS) and negative strand viral 5'-ppp-RNA)] to stimulate primary cultured mouse AEC-IIs. Exosome-based ELISA showed that DAMPs and PAMPs induced mouse AEC-IIs to release CD63-positive exosome in a time-dependent manner (FIGURE 1B). The exosomes were separated and enriched by centrifugal ultrafiltration. Typical exosome particle size and morphology 34 (FIGURE 1C and FIGURE 1D) were observed by transmission electron microscopy (TEM) and nanoparticle tracking analysis (NTA).

Mouse AEC-IIs-derived exosome had overlapping cargo compositions in response to different danger signs: proteomic analysis showed that these exosomes shared 82 (59 upregulated and 23 downregulated) differentially expressed protein cargo (FIGURE 1E and FIGURE S1A-S1D). The cell biological process, cellular component and molecular function of these proteins were examined by Gene Ontology (GO) analysis (FIGURE S1E-S1G). Among them, we are interested in STIMATE because of its high abundance in all types of exosomes. STIMATE loading in mouse AEC-IIs-derived exosomes appears to be MyD88 dependent, as DAMPs or PAMPs fail to induce STIMATE loading into exosomes in MyD88-silenced AEC-IIs (FIGURE S1H). Under the induction of DAMPs or PAMPs, the expression of STIMATE mRNA increased, while the STIMATE protein level was not significantly upregulated (FIGURE 1F and 1G) in cell lysates but was upregulated in mouse AEC-IIs-derived exosomes. Polysome experiments 35 showed that DAMP or PAMP induction did not change the translation efficiency of STIMATE mRNA, as shown in FIGURE S2A -S2D. In addition, the inhibition of the lysosome and proteasome degradation pathways (BafA1 and MG132 inhibitors, respectively) did not significantly increase the protein expression of STIMATE in mouse AEC-IIs lysates (FIGURE S2E and FIGURE S2F). The efficiency STIMATE protein degradation could not explain the differences in the mRNA and protein levels. Therefore, we considered the possibility of mouse AEC-IIs loading STIMATE protein into exosomes under the danger signal of inflammation or injury. Throughout the manuscript, we refer to these mouse AEC-IIs-derived STIMATE-positive exosomes as STIMATE+ ADEs.

To observe the clinical significance of STIMATE+-ADEs in the alveolar cavity, we collected bronchoalveolar lavage fluid (BALF) from airway disease patients. The level of STIMATE+ ADEs was much lower in the BALF of 112 ALI/ARDS (72 infectious cases and 40 traumatic cases) and 44 chronic IPF patients than in the BALF of the healthy controls (23 cases) (FIGURE 1H and FIGURE 1I). Importantly, the level of STIMATE+ ADEs was proportional to the blood oxygen index (PaO2/FiO2) in ALI/ARDS patients (FIGURE 1H). However, STIMATE+ ADEs were a poor prognosis indicator and was inversely proportional to compliance of the respiratory system (Crs) in multiple IFPs patients (FIGURE 1I). To observe the immune status of human TRAMs in BALF, CD11b, Siglec F, CD11C and CD64 were used to sort TRAMs (FIGURE S3A), which express specific transcription key regulators of AM identity and self-renewal (FIGURE S3B) 36. Simultaneously, CD86/CD80 and CD163/CD206 were used to distinguish M1-like TRAMs and M2-like TRAMs (FIGURE S3A). In both ALI/ARDS and IPF patients, the STIMATE+ ADE level negatively corresponded to the proportion of M1/M2-like TRAMs (FIGURE 1J). Seahorse analysis was used to assess the extracellular acidification rate (ECAR) and cellular oxygen consumption rate (OCR) of TRAMs in ALI/ARDS and IPF patients to assess anaerobic glycolysis and mitochondrial respiration, and the results are shown in Figure S3C and Figure S3D. Due to ischemia and hypoxia, TRAMs from ALI/ARDS patients exhibited high anaerobic glycolytic metabolic characteristics and suppressed mitochondrial respiration while TRAMS from IPF showed stabilized oxidative phosphorylation (OXPHOS) energy supply. The distinct metabolic phenotypes of AMs in ALI/ARFS and IPF were significantly perturbed by STIMATE+ ADEs (FIGURE 1K). As shown in FIGURE S3E, the correlation of STIMATE+-ADE with M1/M2 ratio and the corresponding prognosis in reconciling the inflammatory and healing phases, together suggest that STIMATE+ ADE regulates the metabolic patterns of human TRAMs.

### STIMATE+ ADE shortage results in spontaneous lung injury and progressive fibrosis

**Figure 2:**
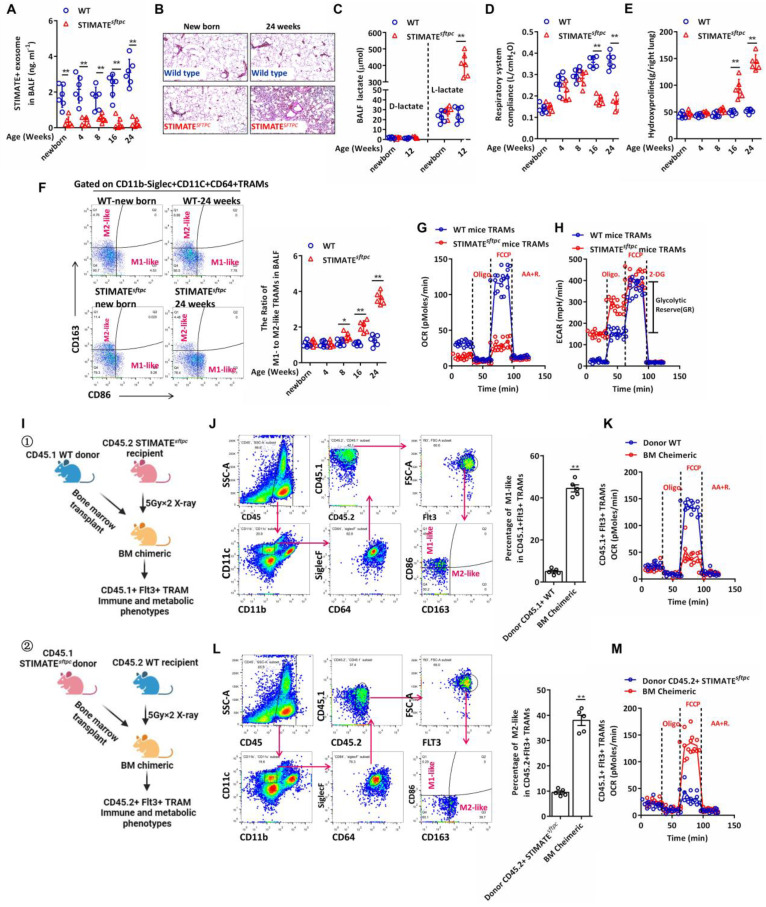
**STIMATE^sftpc^-deficient mice exhibit aseptic spontaneous pulmonary** ***injury and fibrosis. (A)*** The concentration of STIMATE^+^ exosomes in the BALF of WT or STIMATE^sftpc^ mice of different ages was detected by Exo-based ELISA (n = 6). **(*****B)*** H&E staining of paraffin-embedded lung sections from newborn and 24-week-old WT or STIMATE^sftpc^ mice, 400× magnification (n = 6), left lung. **(*****C)*** Plot data based on D-lactic acid and L-lactic acid accumulation in the BALF of WT or STIMATE^sftpc^ mice, n = 6. **(*****D)*** Plot data based on the respiratory system compliance of WT or STIMATE^sftpc^ mice (n = 6). **(*****E)*** Plot data based on hydroxyproline accumulation in the BALF of WT or STIMATE^sftpc^ mice (n = 6). **(*****F)*** Plot data based on the ratio of M1-like to M2-like TRAMs in the BALF of WT or STIMATE^sftpc^ mice (n = 6). ***(G)** In vitro* monitoring of mitochondrial respiration in TRAMs in the BALF of WT or STIMATE^sftpc^ mice, (n = 6). **(*****H)** In vitro* test of glycolysis capacity in TRAMs in the BALF of WT or STIMATE^sftpc^ mice (n = 6). GR: glycolytic reserve. ***(I)***① Schematic diagram of the generation of BM chimeric mice. Lethally irradiated STIMAT-deficient CD45.2^+^ mice received BM transplantation from WT CD45.1^+^ mice, and their TRAMs were mainly derived from Flt3^+^CD45.1^+^ myeloid cells. ② Similarly, TRAMs of recipient CD45.1^+^ WT BM chimeric mice were mainly derived from peripheral Flt3^+^ monocytes of STIMAT^sftpc^ CD45.2 donor mice. **(*****J)*** The sorting strategy of CD45.1*^+^*FLT3*^+^*M1-like TRAMs in type ① BM chimeric mice. The right panel shows data based on CD45.1^+^FLT3^+^M1-like TRAMs in the lungs of BM chimeric mice (n = 5), and CD45.1 +WT donor mice served as controls (n = 5). ***(K)*** The sorting strategy of CD45.2^+^FLT3^+^M2-like TRAMs in type ② BM chimeric mice (n = 5),, and CD45.2^+^ STIMAT^sftpc^ donor mice served as controls (n = 5). ***(L and M)*** Seahorse monitoring of mitochondrial respiration CD45.1^+^FLT3^+^ M1-like and CD45.2^+^FLT3^+^ M2-like TRAMs in type ① and type ② BM chimeric mice, respectively (n = 5). Data are shown as the mean ± SD, *p < 0.05 and **p < 0.01 by Student's t test, analysis of variance (ANOVA) followed by a post hoc test. All cell experiment data were derived from 3 independent experiments.

Next, we constructed the conditional knockout mice STIMATE^flox/flox^; SFTPC-Cre (STIMATE^sftpc^), in which STIMATE was specifically knocked out in mouse AEC-IIs (FIGURE S4A). Compared with WT mice, STIMATE^sftpc^ mice showed completely suppressed STIMATE expression in mouse AEC-IIs at all ages (FIGURE S4B), and the concentration of STIMATE^+^ ADEs in STIMATE^sftpc^ mouse BALF was significantly lower than that in WT mouse BALF (FIGURE 2A). Although reared in a sterile environment, STIMATE^sftpc^ mice developed spontaneous chronic lung injury (FIGURE 2B) accompanied by uncontrolled endogenous L-lactic acid in young mice (FIGURE 2C), and then progressively attenuated respiratory system compliance (FIGURE 2D) and hydroxyproline accumulation in the lungs (FIGURE 2E). More importantly, the proportion of M1/M2-like TRAMs in the BALF of STIMATE^sftpc^ mice decreased significantly compared to that of WT mice (FIGURE 2F). M1-like and M2-like TRAMs had differences in anti- or pro-proinflammatory/profibrotic cytokine release (FIGURE S4C), so their ratios caused a spontaneous immune imbalance in STIMATE^sftpc^ mice. *STIMATE^sftpc^ mouse TRAMs e*xhibited decreased OXPHOS (maximal oxygen consumption capacity of mitochondria after FCCP uncoupling) and glycolytic reserve (GR, defined as the ability to upregulate aerobic glycolysis, following inhibition of mitochondrial ATP synthesis by oligomycin and inner membrane depolarization by FCCP) compared to wild-type mouse TRAMs (FIGURE 2G and FIGURE 2H). These data suggest that STIMATE plays a critical role in maintaining the M1-like immunophenotype and OXPHOS metabolic phenotype of TRAMs.

Lack of STIMATE^+^ ADEs in STIMATE^sftpc^ mice results in phenotype loss of the M2-like TRAMs and metabolic abnormalities. However, two key points still need to be resolved: (1) whether endogenous STIMATE in TRAMs can maintain its M2-like phenotype and (2) whether peripheral monocyte-derived TRAMs are mediated by STIMATE^+^ ADE regulatory phenotype, as TRAMs are contributed by fetal liver monocytes (FL-MOs) 37 and marrow-derived monocytes (BM-MOs) 38. To this end, we designed BM chimeras (FIGURE 2I). Briefly, lethally irradiated CD45.2^+^ STIMATE^sftpc^ mice received BM transplantation from CD45.1 WT donor mice, and after 7 weeks, the AM subpopulation in recipient mice was completely replaced by Flt3^+^ (BM-derived cell-specific marker 39) CD45.1^+^ TRAMs. Flt3^+^ TRAMs from CD45.1^+^ WT donor mice in STIMATE^sftpc^ recipient mice lost their M2-like phenotype and OXPHOS metabolic propensity, even in the presence of endogenously expressed STIMATE in their cells (FIGURE 2J and FIGURE 2K). Likewise, BM-derived TRAMs from CD45.2^+^ STIMATE^sftpc^ mice restored the M2-like immunophenotype and OXPHOS metabolic propensity in the lung microenvironment of CD45.1^+^ WT recipient mice (FIGURE 2L and FIGURE 2M). These data indicate that STIMATE^+^ ADEs control the immune and metabolic phenotypes of TRAMs in the alveolar microenvironment, whether derived from mouse embryonic AEC-IIs or myeloid AEC-IIs. Furthermore, the expression of endogenous STIMATE in TRAMs is not sufficient to maintain their own M2-like phenotype and OXPHOS predisposition.

### Uptake of STIMATE^+^ ADEs enhances TRAM store-operated Ca^2+^ entry (SOCE)-dependent Ca^2+^ influx responsiveness

**Figure 3:**
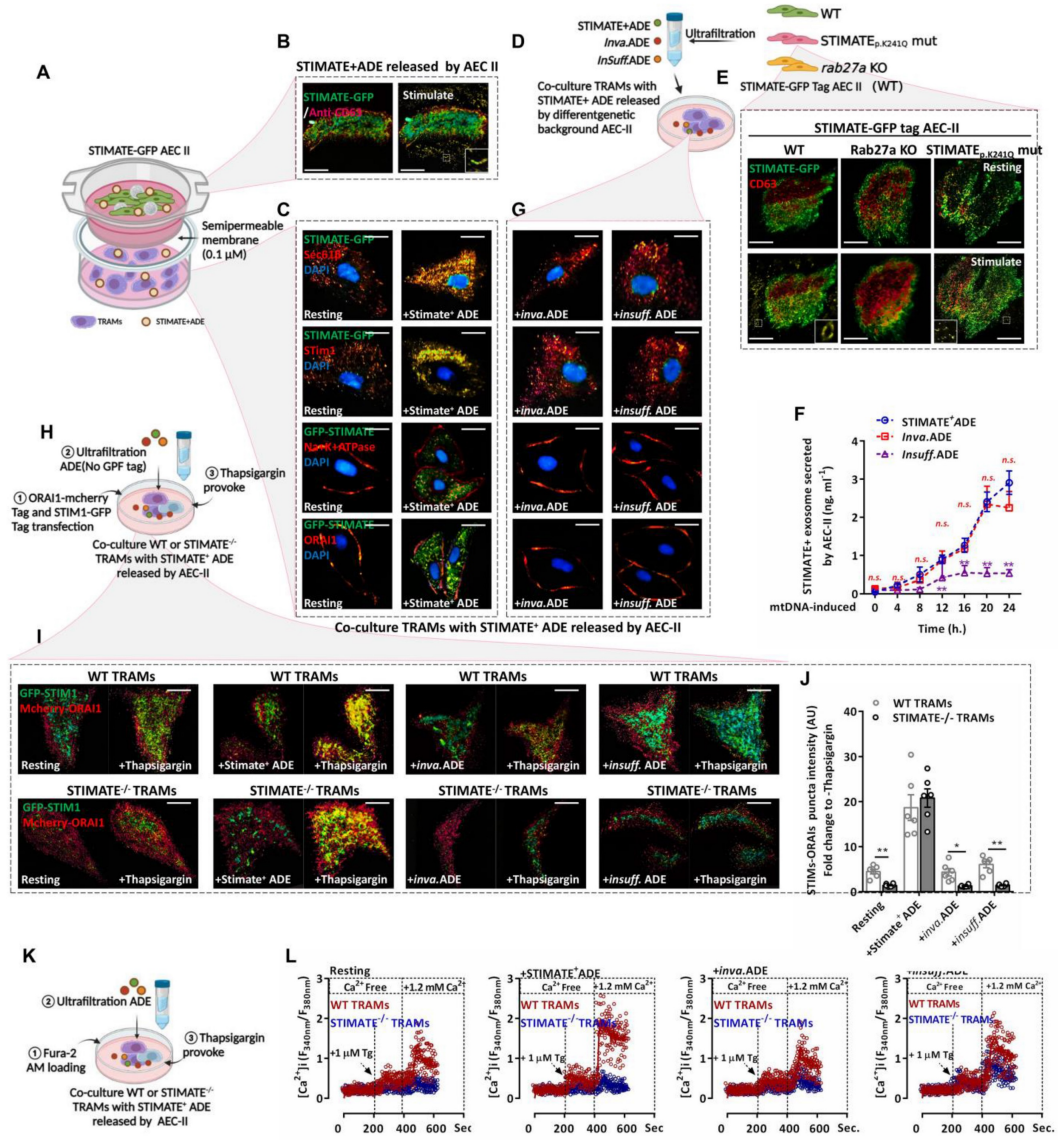
**STIMATE^+^ ADE** ***affects the SOCE-dependent Ca^2+^ influx of TARMs. (A)***Cocultivation model diagram of GFP-STIMATE mouse AEC-IIs with mouse TRAMs in a transwell plate. A 30 kDa semipermeable membrane ensures that exosomes smaller than 100 nm can pass through the pores. **(B)**Representative mouse AEC-IIs TIRF image of GFP-STIMATE^+^ exosomes in the transwell top chamber after transfection with mtDNA after 24 h. **(C)**Representative CLSM of mouse TRAMs in the transwell bottom chamber after 12 h of cocultivation. The images from top to bottom sequentially show the merged GFP-STIMATE with anti-Sec61β (ERM marker), anti-STIM1, anti-Na^+^Ka^+^ ATPase (PM marker) and anti-ORAI1. Scale bar: 2 μm. **(D)**The genetic background generated by STIMATE^+^-ADE, Inva.ADE and Insuff.ADE was secreted by GFP-tagged STIMATE WT AEC-IIs, STIMATE^P. K241Q^ AEC-IIs and Rab27a KO mouse AEC-IIs. **(E)**Representative mouse AEC-IIs TIRF image of STIMATE^+^-ADE, Inva.ADE and Insuff.ADE after transfection with mtDNA after 24 h. Scale bar: 2 μm. **(F)**exosomes-based ELISA detects STIMATE^+^-ADE, Inva.ADE and Insuff.ADE released by GFP-tagged STIMATE WT AEC-IIs, STIMATE^P. K241Q^ AEC-IIs and Rab27a KO mouse AEC-IIs after transfection with mtDNA after 24 h, respectively. Draw according to the reorganized STIMATE standard curve (n = 6). **(G)**Representative CLSM of mouse TRAMs in the transwell bottom chamber after 12 h of cocultivation with Inva.ADE and Insuff.ADE. The images from top to bottom sequentially show the merged GFP-STIMATE with anti-Sec61β, anti-STIM1, anti-Na^+^Ka^+^ ATPase and anti-ORAI1. Scale bar: 2 μm. **(H)**Schematic diagram of the observations of SOCE based on STIM1-ORAI1 colocalized puncta in WT or STIMATE^-/-^ mouse TRAMs after cocultivation with ADE. ***(I and J)*** Representative TIRF image of STIM1-GFP tag/ORAI1-mCherry tag cotransfected WT or STIMATE^-/-^ mouse TRAMs stimulated with 1 μM TG for 8 min after cocultivation with STIMATE^+^-ADE, Inva.ADE and Insuff.ADE for 12 h, sequentially. The bar graph on the right shows the fluorescence density values of the fused fluorescent spots (n = 6). Scale bar, 2 μm. ***(K)*** Schematic diagram of the observations of Ca^2+^ influx based on SOCE in WT or STIMATE^-/-^ mouse TRAMs after cocultivation with ADE. ***(L)*** Ca^2+^ influx in WT or STIMATE^-/-^ mouse TRAMs preincubated with STIMATE^+^-ADE, Inva.ADE and Insuff.ADE before 1 μM TG induction. The figure shows the ratio of excitation wavelengths of Fura-2 AM at 340 nm and 380 nm. for n = 7-9 cells pooled across 3 independent experiments. Data are shown as the mean ± SD, *p < 0.05, and**p < 0.01 by analysis of variance (ANOVA) followed by a post hoc test compared to resting TRAMs. *All data were derived from 3 independent experiments.*

To observe Exo-based crosstalk between AEC-IIs and TRAMs, the coculture protocol is shown in FIGURE 3A. In addition, we constructed mouse AEC-IIs with GFP-tagged STIMATE (FIGURE S5A and FIGURE S5B) to track STIMATE^+^ ADE uptake. Total internal reflection fluorescence (TIRF) imaging showed that mouse AEC-IIs can release GFP-tagged STIMATE^+^-ADE fluorescent particles (FIGURE 3B). STIMATE is a positive regulator of SOCE-dependent Ca^2+^ influx located in the endoplasmic reticulum membrane (ERM) 40. GFP-tagged STIMATE protein was also detected in TRAMs in the transwell bottom chamber and colocalized with STIM1 on the ERM (Sec61β marker). In contrast, ORAI1, the control, on the cytoplasmic membrane (PM) (Na/K ATPase marker) did not bind with the GFP tag STIMATE protein (FIGURE 3C).

Next, we constructed mouse AEC-IIs in two genetic backgrounds (FIGURE 3D). The STIMATE^p.K241Q^ mutation was obtained through site-directed mutagenesis; this mutation disrupted the ER-binding transmembrane domain of STIMATE and blocked STIMATE localization on ERM but did not affect STIMATE expression, making AEC-IIs-generated invalid STIMATE^+^ ADEs (Inva.ADE). Furthermore, CRISPR/Cas9 was used to abrogate Rab27a (a regulator of Exo secretion 41), which resulted in insufficient AEC-II STIMATE^+^ ADE (Insuff.ADE) generation (FIGURE 3E-3F and FIGURE S6A-S6I). As shown in FIGURE 3G and FIGURE S7A-S7C, STIMATE^+^ ADEs under both genetic backgrounds could not be taken up and localized to the ERM of mouse TRAMs. *SOCE-dependent Ca^2+^ influx* is mediated by ORIA1 located in the PM and STIM1 located in the ERM. When Ca^2+^ storage in the ER is exhausted, STIM1 is activated as a Ca^2+^ sensor and connected to ORA1 located in the PM to facilitate SOCE, allowing external Ca^2+^ to enter the ER and the cytoplasm *42-44*. Thapsigargin (TG) depletes ER Ca^2+^ by inhibiting sarco/endoplasmic reticulum Ca^2+^ ATPase (SERCA) and then induces SOCE 45. TIRF microscope imaging showed that under TG excitation, STIM1 and ORIA1 formed puncta and tightly colocalized in mouse TRAMs, and preincubation with STIMATE^+^ ADEs increased STIM1-ORIA1 colocalized puncta, which was abolished in STIMATE^-/-^ mouse TRAMs and was hardly observed in WT mouse TRAMs cocultured with Inva.ADE and insuff.ADE genetic backgrounds (FIGURE 3H-3I).

As a second messenger, the Ca^2+^ entry channel perturbs the energy metabolism and polarization of macrophages 46. To observe the effect of STIMATE^+^ ADEs on the SOCE-Ca^2+^ signal in TRAMs, Fura-2-AM was used to label intracellular Ca^2+^ to observe the transient Ca^2+^ influx due to SOCE (FIGURE 3K). As shown in FIGURE 3L and FIGURE S8A-8C, in the mouse TRAMs preincubated with STIMATE^+^ ADEs, the peak of TG-activated transient Ca^2+^ influx was stronger than that of the untreated TRAMs, and SOCE-dependent Ca^2+^ influx was abolished in STIMATE^-/-^ mouse TRAMs. Furthermore, no significant transient Ca^2+^ influx change was observed in TRAMs cocultured with inva.ADE or Insuff.ADE.

Therefore, STIMATE is essential for maintaining the SOCE-dependent Ca^2+^ influx of TRAMs, while exogenous STIMATE^+^ ADEs may modulate the immune status and metabolic predisposition of TRAMs by controlling the responsiveness of Ca^2+^ influx.

### STIMATE^+^ ADEs control the metabolic switch of mouse TRAMs

**Figure 4:**
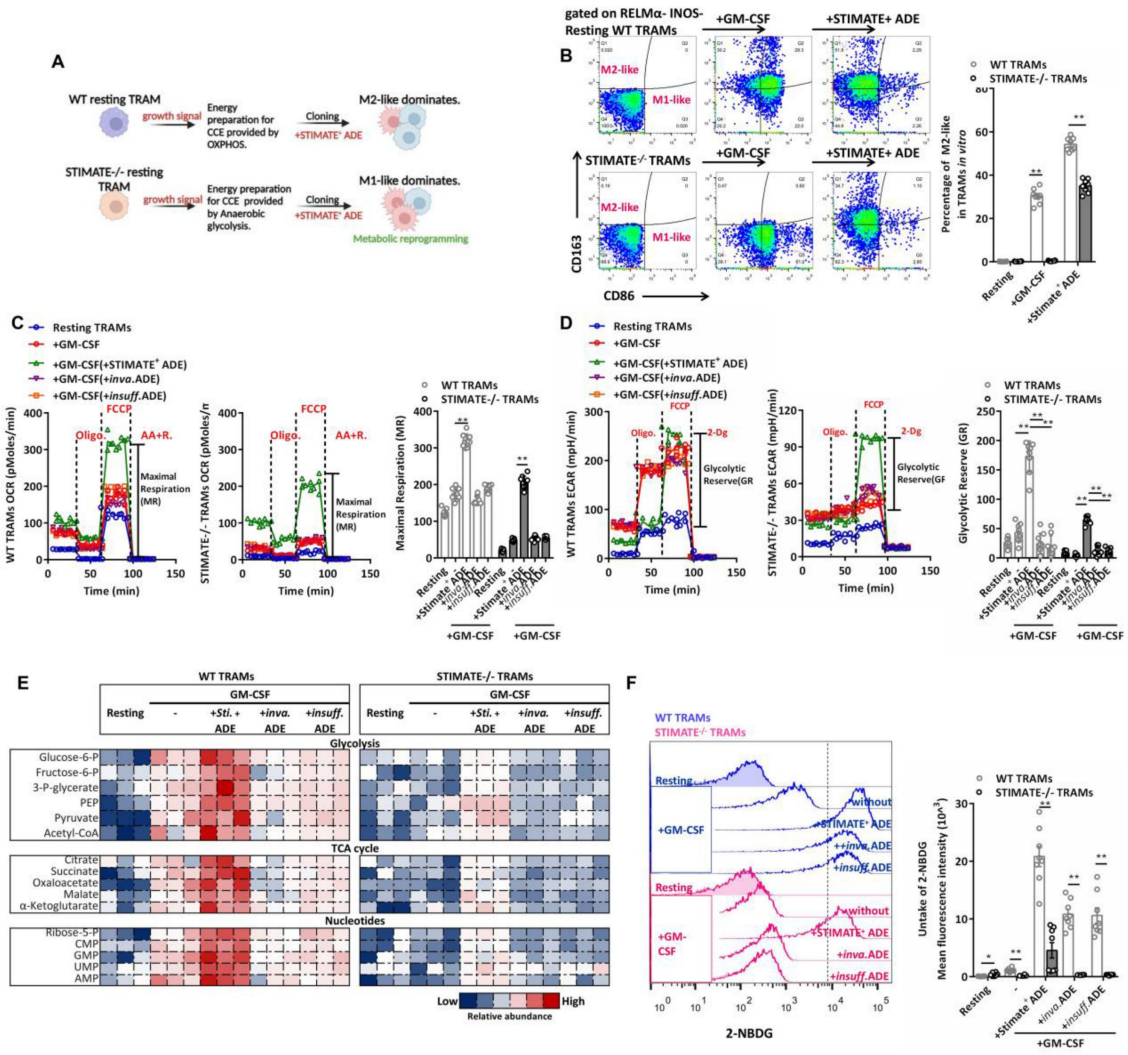
** STIMATE^+^ ADE affects TRAMs immune selection and perturbs glycolysis and the Krebs cycle.** ***(A)*** Schematic diagram of the TRAMs immunophenotypic selection following CCE and metabolic switching controlled by STIMATE^+^ ADE. **(B)** Flow cytometry analysis of M1-like and M2-like WT or STIMATE^-/-^ mouse TRAMs induced by GM-CSF after cocultivation with STIMATE^+^ ADE for 12 h (n = 8). **(C)** Seahorse monitoring of mitochondrial respiration in WT or STIMATE^-/-^ mouse TRAMs induced by GM-CSF after cocultivation with STIMATE^+^ ADE, Inva.ADE and Insuff.ADE for 12 h (n = 3). MR: maximal respiration. ***(D)*** Seahorse test of glycolysis capacity in WT or STIMATE^-/-^ mouse TRAMs induced by GM-CSF after cocultivation with STIMATE^+^-ADE, Inva.ADE and Insuff.ADE for 12 h (n = 3). GR: glycolytic reserve. **(E)** The abundance of polar metabolites of the tricarboxylic acid cycle and the glycolytic pathway in WT or STIMATE^-/-^ mouse TRAMs induced by GM-CSF after cocultivation with STIMATE^+^ ADE, Inva.ADE and Insuff.ADE for 12 h (n = 3). Heatmap showing log normalized values from minimum (blue) to maximum (red). **(F)** Flow cytometry analysis of the fluorescence intensity of glucose mimetic 2-NBDG uptake in WT or STIMATE^-/-^ mouse TRAMs induced by GM-CSF after cocultivation with STIMATE^+^ ADE, Inva.ADE and Insuff.ADE for 12 h (n = 8). Data are shown as the mean ± SD, *p < 0.05, and**p < 0.01 by analysis of variance (ANOVA) followed by a post hoc test compared to resting TRAMs. *All data were derived from 3 independent experiments.*

The immune selection of TRAMs is driven by metabolic phenotypes, which in turn are involved in both danger and growth signals in the microenvironment. To evaluate whether STIMATE^+^ ADEs perturb the long-term Ca^2+^ signal transduction of TRAMs cell cycle entry (CCE), which has a significant impact on gene expression and epigenetic regulation of TRAMs 47, we observed the selection of immune and metabolic phenotypes in resting phase mouse TRAMs (i.e., RELMα^low^INOS^low^ TRAMs) stimulated by growth signals (FIGURE 4A). Long-term Ca^2+^ influx accumulation under GM-CSF stimulation is still subject to transcellular regulation by STIMATE^+^ ADEs (FIGURE S9A), which is determined by STIMATE-mediated long-term maintenance and remodeling of ERM-PM junctions (FIGURE S9B).

CCE of mouse TRAMs was controlled by STIMATE and recovered with STIMATE^+^ ADE supplementation (FIGURE S9C). The M1-like (FIGURE 4B) and RELMα^low^ INOS^hi^ (FIGURE S9D-9E) phenotypes were more common in STIMATE^-/-^ mouse TRAMs. In contrast, the M2-like immune selection of RELMα^hi^ INOS^low^ was more common in WT mouse TRAMs (FIGURE 4B and FIGURE S9D-9E). This suggests that endogenous STIMATE is not sufficient to support the dominant immunoselection of resting mouse TRAMs to M2-like, but if STIMATE is completely abolished, resting mouse TRAMs will transition to M1-like. In terms of energy metabolism patterns, STIMATE^-/-^ mouse TRAMs stimulated by growth signals had lower mitochondrial respiration capacity and lower GR, suggesting that they prefer to utilize anaerobic glycolysis as an energy supply for growth in contrast to the OXPHOS energy supply used by WT mouse TRAMs (FIGURE 4C-4D). Importantly, coculture of STIMATE^+^ ADEs allowed STIMATE^-/-^ mouse TRAMs to restore OXPHOS energy patterns and M2-like immunophenotypic selection (FIGURE 4C-4D). The abundance of polar metabolites was observed by LC-MS. As expected, the Krebs cycle and the glycolytic pathway in STIMATE^-/-^ mouse TRAMs became fragmented as respiration declined, while in STIMATE^+^ ADE cocultures, the disrupted Krebs cycle and glycolytic pathways were rejuvenated (FIGURE 4E). In addition, the production of nonessential and essential amino acids was also affected by STIMATE^+^ ADEs (FIGURE S10A), which is important for GM-CSF-induced TRAMs CCE.

STIMATE deficiency interferes with the uptake of glucose (2-NBDG fluorescent glucose mimetic) by TRAMs (FIGURE 4F), as restricted STIMATE affects the expression of the glucose transporters Glut1 and Glut3 (FIGURE S10B-S10C). However, STIMATE^+^ ADEs can restore and enhance glucose uptake efficiency and Glut1/3 expression in TRAMs. These data suggest that STIMATE^+^ ADEs not only affect the transient Ca^2+^ influx responsiveness of TRAMs but also likely transcellular regulate the metabolic switch of TRAMs during immune selection in a longer-lasting manner through reprogramming.

### STIMATE^+^-ADE controls mitochondrial biogenesis and ETC-mtDNA coding

**Figure 5:**
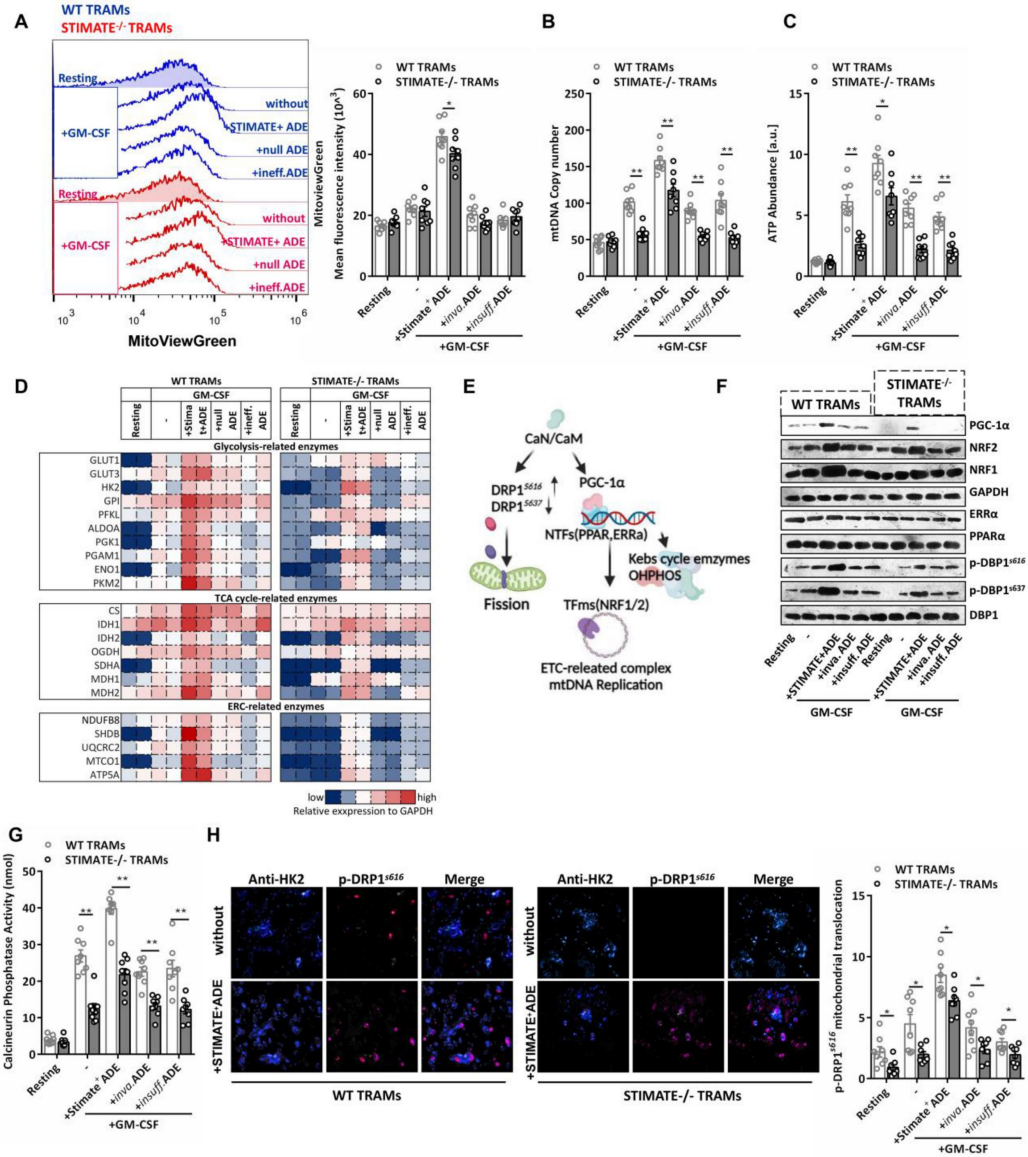
** STIMATE^+^ ADE affects the TRAM CaN-PGC-α pathway and** ***mitochondrial biogenesis. (A)*** Flow cytometry analysis of the mitochondrial volume by MitoViewGreen Fluorescence staining in WT or STIMATE^-/-^ mouse TRAMs preincubated with STIMATE^+^ ADE, Inva.ADE and Insuff.ADE for 12 h before rmGM-CSF induction (n = 8). The right figure shows the MFI (495 nm absorption wavelength/516 nm emission wavelength) quantification of MitoViewGreen in TRAMs. **(B and C)** mtDNA copy number and ATP abundance by qPCR and LC-MS in WT or STIMATE^-/-^ mouse TRAMs preincubated with STIMATE^+^ ADE, Inva.ADE and Insuff.ADE for 12 h before rmGM-CSF induction (n = 8). **(D)** The expression of key glycolytic metabolic enzymes, TAC enzymes and ETC enzymes in WT or STIMATE^-/-^ mouse TRAMs by prefabricated custom antibody arrays and preincubated with STIMATE^+^-ADE, Inva.ADE and Insuff.ADE for 12 h before rmGM-CSF induction (n = 2). The panel is the heatmap drawn after normalizing the fluorescence intensity data of each point. **(E)** Schematic diagram of the involvement of PGC-1α and CaN in mitochondrial biogenesis, mitochondrial DNA replication, and mitochondrial fission. ***(F)*** Representative western blotting images of PGC-1α, PPARα, ERRα, NRF1 and NRF2 expression in WT or STIMATE^-/-^ mouse TRAMs preincubated with STIMATE^+^-ADE, Inva.ADE and Insuff.ADE for 12 h before rmGM-CSF induction. GAPDH was used as an internal reference. Phosphorylated DRP1^S616^ and p-DRP1^S637^ were also detected, with DRP1 as an internal control. ***(G)*** Standard colorimetric assay to detect CaN enzyme activity in WT or STIMATE^-/-^ mouse TRAMs preincubated with STIMATE^+^-ADE, Inva.ADE and Insuff.ADE for 12 h before rmGM-CSF induction (n = 8). **(H)** Representative CLSM of the recruitment of DRP1^s616^ to the mitochondrial outer membrane (anti-HK2) in WT or STIMATE^-/-^ TRAMs preincubated with STIMATE^+^-ADE, Inva.ADE and Insuff.ADE for 12 h before rmGM-CSF induction (n = 8). The bar graph shows the quantification of the indicated number of cells showing DRP1^s616^ translocation to the mitochondria. Scale bar, 1 μm. Data are shown as the mean ± SD, *p < 0.05 and **p < 0.01 by analysis of variance (ANOVA) followed by a post hoc test compared to resting TRAMs*.* All data were derived from 3 independent experiments.

In the mouse TRAMs pretreated with STIMATE^+^ ADEs, GM-CSF increased the mitochondrial volume by approximately 2.5 times (FIGURE 5A) and was accompanied by an increase in the number of copies of mtDNA (FIGURE 5B) and the abundance of intracellular ATP (FIGURE 5C), which indicates that STIMATE^+^ ADEs are involved in mitochondrial biogenesis. Prefabricated custom antibody arrays showed that STIMATE^+^ ADEs enhanced the expression of glycolytic pathway-related and OXOHOS-related metabolic enzymes during TRAMs expansion, especially the key components of the mitochondrial electron respiratory chain (ERC) complex. The expression levels of NDUF88, SHDB, UQCRC2, MTCO1 and ATP5A were enhanced (FIGURE 5D and FIGURE S10D). Despite being a smaller component of the genome, mtDNA encodes a series of mitochondrial ERC-related enzymes and is driven by the nuclear-encoded mitochondrial transcription factor A (Tfam) promoter *48*. The Tfam promoter contains recognition sites for NRF1 and/or NRF2, is transcriptionally initiated by ERRα and PPARα and is coregulated by PGC-1α as a transcriptional partner 49, thus allowing coordination between mitochondrial and nuclear activation during mitochondrial biogenesis (FIGURE 5E).

STIMATE^+^ ADEs enhanced the protein expression of NRF1, NRF2 and PGC-1α during clone expansion of mouse TRAMs but had no effect on the expression of ERRα and PPARα (FIGURE 5F). The PGC-1α promoter luciferase reporter plasmid (FIGURE S11A-S11B), Co-IP assay (FIGURE S11C) and PGC-1α translocation into nucleus confocal assay (FIGURE S11D) together showed that STIMATE^+^ ADEs can enhance PGC-1α interaction with ERRα and PPARα and coactivate NRF1 and NRF2 expression, which is important for ETC-related enzymes coding for mtDNA. PGC-1α has various posttranscriptional regulatory and posttranslational modifications 50, the most important of which is the acceptance of transcriptional regulation from calcineurin (CaN) and Ca^2+^-calmodulin-dependent kinase (CaMK) 51-52. The enzymatic activity of CaN in mouse TRAMs is regulated by STIMATE and affected by STIMATE^+^ ADEs (FIGURE 5G). Interestingly, dynamo-related protein (DRP1), which drives mitochondrial fission, is also activated by CaN by phosphorylating the S616 site 53. In the ensuing observations, STIMATE^+^ ADEs enhanced p-DRP1 s616 phosphorylation and decreased p-DRP1 s637 phosphorylation (FIGURE 5F) and controlled p-DRP1 s616 recruitment to mitochondria (FIGURE 5H). These data suggest that STIMATE^+^ ADEs regulate the metabolic switching of mouse TRAMs through CaN activity and PGC-1α costimulation. Further experiments showed that the intracellular Ca^2+^ chelator BAPTA-AM, the CaN inhibitor tacrolimus (FK506) and cyclomycin A (CsA) as well as PGC-1α shRNA all blocked mouse TRAMs glucose utilization (FIGURE S12A-S12C) and mitochondrial biosynthesis (FIGURE S13A-S13D). These data suggest that STIMATE^+^ ADEs control mitochondrial biogenesis and ETC-mtDNA coding and mitochondrial fission via CaN-PGC-1α signaling and DRP1 phosphorylation of DRP1, respectively.

### Therapeutic STIMATE^+^ ADEs are a potential alternative to stem cell transplantation in pulmonary fibrosis

**Figure 6:**
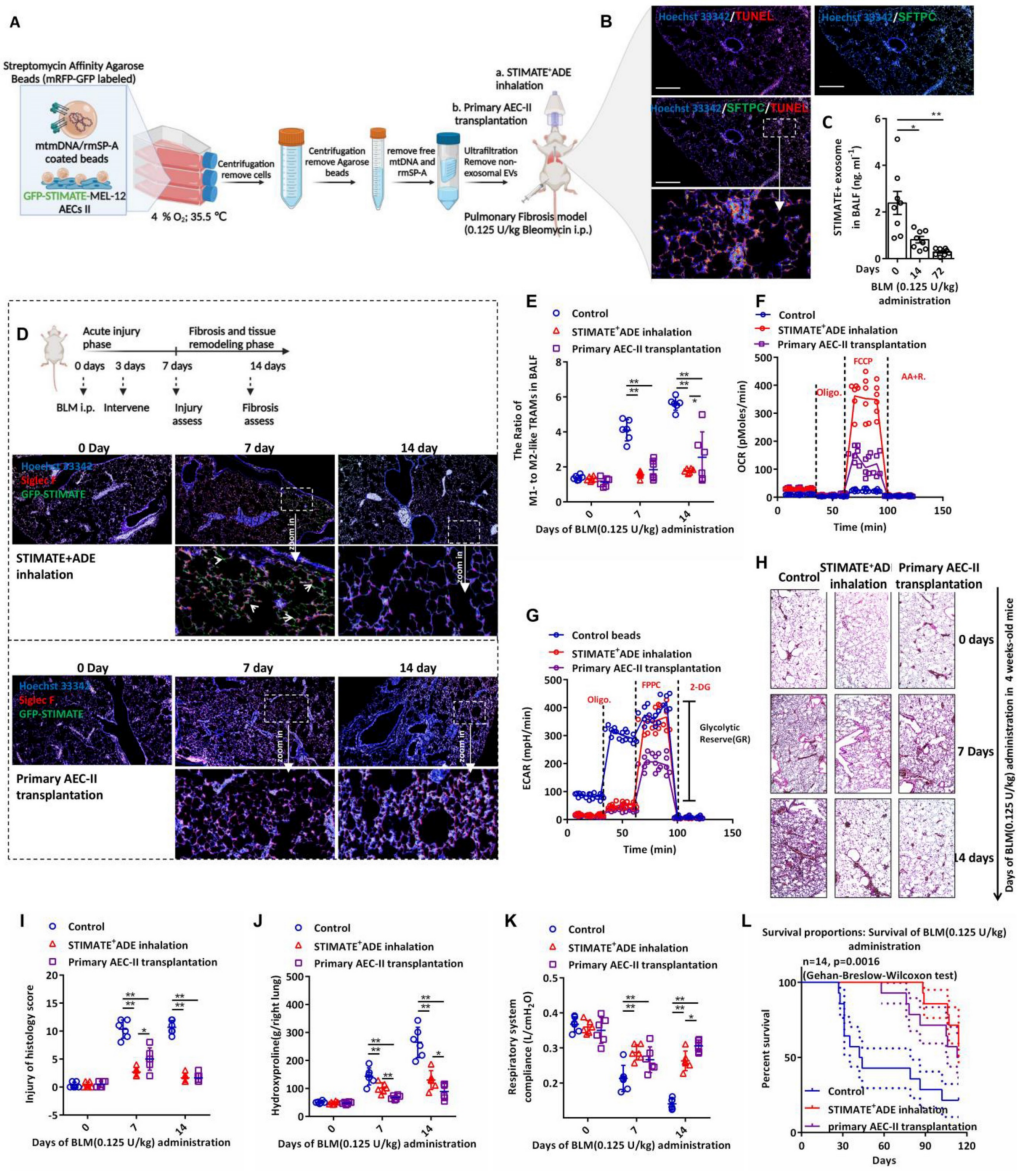
***Therapeutic STIMATE^+^ ADE in the BLM-induced injurious pulmonary fibrosis model. (A)*** The protocol of rmSP-A/mtDNA-coated agarose beads stimulate type II alveolar epithelial MLE-12 cells to secrete sufficient STIMATE*^+^* ADE for translational treatment of BLM-induced pulmonary fibrosis. ***(B)*** Confocal images of apoptosis-labeled TUNEL and mouse AEC-IIs-labeled SFTPC of BLM-induced mouse lung sections after 14 days of induction with 0.125 U/kg BLM. **(C)** ELISA detected the level of STIMATE^+^-ADE in the BALF of mice after 14 days of induction with 0.125 U/kg BLM (n = 8). ***(D)*** Mouse lung immunofluorescence sections fluorescent merge of Siglec F-labeled AMs and STIMATE-GFP ADE. Administration of inhaled atomized particles to mice (twice a week, 10^9^ to 10^10^ particles per mouse) and mouse AEC-IIs tracheal colonization (2×10^6^ cells per mouse) after intraperitoneal administration of 0.125 U/kg bleomycin. **(E)** Flow cytometry to detect the ratio of M1-like and M2-like TRAMs in mouse BALF after intraperitoneal administration of 0.125 U/kg bleomycin, preadministration of inhaled atomized particles to mice or mouse AEC-IIs tracheal colonization (n = 6). **(F and G)** Seahorse monitoring of mitochondrial respiration and glycolysis capacity in BALF TRAMs after 7 days and 14 days of BLM intraperitoneal administration in the presence or absence of inhaled atomized particles to mice or mouse AEC-IIs tracheal colonization (n = 6). **(H and I)** H&E staining and a pathological score of paraffin-embedded lung sections after 7 days and 14 days of BLM intraperitoneal injection in the presence or absence of inhaled atomized particles to mice or mouse AEC-IIs tracheal colonization. 400× microscope (n = 6), whole lung. ***(J and K)*** Hydroxyproline content (right lung) and respiratory system compliance of the lung after 7 days and 14 days of BLM intraperitoneal injection in 4-week-old mice in the presence or absence of inhaled atomized particles to mice or mouse AEC-IIs tracheal colonization (n = 6). ***(L)*** BLM induces long-term survival in mice with chronic pulmonary fibrosis in the presence or absence of inhaled atomized particles or mouse AEC-IIs tracheal colonization (n = 14). Data are shown as the mean ± SD, *p < 0.05 and **p < 0.01 by paired Student's t test and Gehan-Breslow-Wilcoxon test (FIGURE W) compared to WT mice or untreated mice.

Considering the stem cell properties of AEC-IIs and their potential as a source of therapeutic exosomes, we next evaluated the therapeutic effect of STIMATE^+^ ADEs in a lung fibrosis mouse model. Nebulized STIMATE^+^ ADE inhalation for the treatment of *bleomycin* (BLM)-induced AEC-IIs injury model and the treatment protocol are shown in FIGURE 6A. As expected, BLM induced extensive apoptosis of mouse AEC-IIs (FIGURE 6B), which resulted in abolished secretion of endogenous STIMATE^+^ ADEs (FIGURE 6C). Since primary cultured mouse AEC-IIs senesced within 48 to 72 h* in vitro*, we evaluated the potential of the MLE-12 cell line as a source of therapeutic exosomes. The MLE-12 cell line is an SV40 T virus-immortalized cell line with stable AEC-II properties. MLE-12 cells secreted a large amount of STIMATE^+^ ADEs when stimulated by DAMPs or PAMPs (FIGURE S14A). Among them, mtDNA transfection could stimulate MLE-12 cells to produce STIMATE^+^ ADEs at a concentration of up to 10 ng ml^-1^ (this is a 5-fold change from that detected in BALF). Low oxygen (FIGURE S14B) and low temperatures (FIGURE S14C) improved the STIMATE^+^ ADE yield, but high lactate environment did not (FIGURE S14D).

mtDNA is the recognition ligand of intracellular RIG-I and MDA5 receptors 54; meanwhile, TLR7/TLR9 in endolysosomes can also recognize mtDNA and induce a strong IFN-I response *55*. Considering that epithelial cells can perform phagocytosis and that SP-A can mediate epithelial endocytosis through *clathrin 56*, we designed rmSP-A/mtDNA-coated agarose beads to stimulate clathrin-dependent endocytosis (CDE) of MLE-12 cells. As shown in FIGURE S14E, rmSP-A/mtDNA-coated beads were taken up by MLE-12 cells and delivered to endolysosomes (yellow mRFP-GFP was degraded to red mRFP in acidic endolysosomes). The extracellular domain of TLR9/TLR7 is cleaved in the acidic compartment of the endolysosome, and only the cleaved form of TLR9/TLR7 recognizes mtDNA and recruits and activates MyD88 signaling 55. As shown in FIGURE S14F, rmSP-A/mtDNA-coated agarose beads enabled the cleavage and activation of TLR7/TLR9 in isolated endolysosomes. Accompanied by hypoxia (4% O_2_), lactate-free and sublow temperature (35.5 ℃), the yield of STIMATE secreted by MLE-12 cells was approximately 16.7-fold higher than that of basal secretion (FIGURE S14G), and sufficient number of exosomes could be obtained for *in vivo* administration. The exosomes secreted by MLE-12 cells induced by rmSP-A-mtDNA beads produced the same characteristic protein cargo as the primary mouse AEC-IIs induced by mtDNA (FIGURE S16A). Importantly, compared with the direct transfection of mtDNA, rmSP-A-mtDNA beads resulted in exosome products that contained extremely low levels of free mtDNA contamination (FIGURE S16B-16E), and elimination of these pyrogens contributes to the safety of exosomes administered *in vivo*.

To evaluate the difference in the intervention effect of AEC-IIs tracheal colonization and atomized STIMATE^+^ ADE inhalation in BLM-induced acute injury and chronic fibrosis (FIGURE 6D), STIMATE^+^ ADE or mouse AEC-IIs transplantation intervention was performed on days 3 after BLM intraperitoneal administration, and acute lung injury and fibrosis assessed on the following day 7 and day 14, respectively. GFP-STIMATE^+^ ADEs localized within individual cells positive for Siglec F, a biomarker for AMs, 4 days after inhalation administration. After 11 days, STIMATE^+^ ADEs were completely degraded, which slowed down the exacerbation of fibrosis that might be caused by M2-like TRAMs. The shortage of STIMATE^+^ ADEs makes it difficult to restore M1/M2-TRAMs to physiological ratios (FIGURE 6E), and prone to weakening the OXPHOS and GR metabolic phenotypes (FIGURE 6F-6G). Administration of inhaled atomized particles to mice (10^9^ to 10^10^ particles per mouse) and mouse AEC-IIs tracheal colonization (2×10^6^ cells per mouse) maintained a stable ratio of M1- to M2-TRAMs and shifted the metabolic state of weakened OXPHOS to lower GR in mouse TRAMs induced by BLM (FIGURE 6E-6G). The STIMATE^+^ ADE inhalation group showed better early injury relief (FIGURE 6H and FIGURE 6I), while mouse AEC-IIs tracheal colonization prevention was better in slowing hydroxyproline deposition (FIGURE 6J). As a result, both STIMATE^+^ ADE inhalation and mouse AEC-IIs tracheal colonization can ameliorate respiratory compliance and tension and reduced the death rate of BLM-treate mice (FIGURE 6K-6L).

These data indicate that STIMATE^+^ ADEs maintain a steady metabolic state and control the proportion of M1- and M2-TRAMs and are a possible alternative to AEC-IIs transplantation into the airway.

## Discussion

As they form a direct barrier for blood-gas circulation, AEC-IIs are more susceptible to endogenous infection or exogenous injury, such as high expression of ACE2, making it a major target for COVID-19 infection and rapidly developing into ARDS 57. In interstitial PF, extensive AEC-IIs impairment leads to progressive lung compliance deterioration and gas exchange failure 58. AECs have a vigorous exosomes secretion capacity and participate in intercellular communication by carrying RNAs, enzymes, or proteins 22,59-60. We observed that AEC-IIs released more than 10 times the baseline level of CD63^+^ exosomes within 24 h after stimulation by the danger signals PAMPs and DAMPs. These exosomes share a variety of protein cargos, such as SOCS3 and CASP3, which have been found in BALF exosomes and are involved in airway diseases 59,61. Among them, STIMATE is loaded efficiently into secreted exosomes and taken up by TRAMs.

The loading of STIMATE into ADEs seems to be myD88 dependent, which explains how different risk signals stimulate the secretion of STIMATE*^+^* ADE. Long-term studies have shown that LPS-induced TLR4 activation of intracellular signals transmitted by MyD88, while TLR7/9 signaling depends on MyD88 recruitment to the lysosome. The signal transduction of NF-kB by HMGB1 is also assisted by myD88 62. However, the mechanism of how myD88 regulates the loading of stimulus into ADE seems to be complex and limited by space, which we will explore in future studies. The reduced concentration of STIMATE-positive ADEs in the lungs of mice with AEC-IIs conditional knockout of STIMATE was 87%, which indicates that AEC-IIs are the main source of STIMATE^+^ exosomes. Even under sterile conditions, STIMATE^sftpc^ mice spontaneously developed chronic lung injury, fibrosis, and ventilation disorder, which is associated with anaerobic glycolysis of excess M1-like TRAMs and the resulting endogenous lactate environment. The phenotype of irreparable STIMATE^+^-ADE of STIMATE^sftpc^ mice is reminiscent of ALI/ARDS characterized by excessive inflammatory cytokine release, diffuse epithelial cell damage and progressive fibrosis hyperplasia 58,63. More importantly, the STIMATE^+^ ADE level was significantly lower in the BALF of ALI/ARDS patients (112 cases) than that of healthy subjects (23 cases) and was negatively correlated with the severity of hypoxia, and high STIMATE^+^ ADE represents a poor prognosis in patients with advanced fibrosis. A more pronounced trend was observed in patients with interstitial pulmonary fibrosis (44 cases). This clinical finding evokes the hypothesis that timely supplementation with STIMATE^+^-ADE can rescue injury, delay the progression of fibrosis, and restore ventilatory disorders. Natural exosomes have been used as a therapeutic vector and have great advantages compared to traditional drugs 64-65, and the property that AEC-II derivatives also ensure that they can be efficiently delivered into the interior of the alveoli without being hindered by pulmonary surfactant.

In the lung, nearly all cell types can re-enter the cell cycle and exhibit some motility to facilitate barrier reconstruction, and in the damaged lung parenchyma, residual AEC-IIs from distal airways/progenitor cells contribute to alveolar repair *66*. Stem cell transplantation of AEC-IIs is very attractive for the treatment of lung disorders 67. To date, the main sources of AEC-IIs are donors, patient residual primary cell cultures and autologous induced pluripotent stem cell (iPSCs)-directed differentiation. The average survival time of primary AEC-IIs *in vitro* is 48-72 h limits their use as a stable source of donors. *In vitro* induction of hPSCs into differentiated AEC-IIs-like progenitor cells has great therapeutic potential *66-67,* and the combination of hiPSCs for the rapid and economical production of AEC-IIs-derived exosomes has a very attractive prospect. Recent studies have reduced the genetic risk and complex induction procedures of hiPSCs^68^-^69^. SP-A is related to the surfactant recycling by AEC-IIs, the interaction between SP-A and AEC-IIs includes covers a wide range of biological effects, including the regulation of surfactant lipid turnover and modulation of cytokine release 56. We utilized SP-A to mediate MLE-12 endocytosis, which promoted the formation of mtDNA endolysosomes and the cleavage of TLR7/TLR9, resulting in a more than 16-fold yield of STIMATE^+^ ADEs compared to the basal secretion of resting MLE-12. The administration of atomized STIMATE^+^ ADE particles twice a week significantly improved the M1-like/M2-like TRAMs imbalance, ventilatory disturbance, chronic damage and fibrosis in BLM-induced model mice and significantly prolonged the survival rate of pulmonary fibrosis mice. Compared with primary AEC-IIs bronchial colonization, STIMATE is more effective at alleviating early lung injury and prolonging mouse survival. Importantly, STIMATE^+^ ADEs could improve the energy utilization pattern of TRAMs by switching from anaerobic glycolysis to OXPHOS, which is crucial for the maintenance of the ratio of TRAMs in the acidic alveolar microenvironment.

AMs reside in the terminal airways and play an essential role in lung development, immune defense, and surface-active substance secretion 72. It was believed that AMs were derived from BM-MO supplements in earlier research 38. In fact, similar to many tissue-resident macrophages, such as microglia, Langerhans cells and Kupffer cells 37, 73-74, AMs have been confirmed to be derived from yolk sac-derived macrophages (YS-Macs) 16,39 and from fetal liver monocytes (FL-MOs)^75^. FL-MOs, as the precursor of alveolar TRAMs, migrate into the lungs before birth and develop into mature long-lived AMs one week after birth 57. The binary M1/M2 macrophage concept previously proposed considers M1 macrophages to be proinflammatory, relying on glycolytic metabolism, while M2 macrophages are efferocytic macrophages with antifibrolast activity that are dependent on OXPHOS 15. Although this dichotomy is now being challenged by the overlapping macrophage spectrum, it has become a consensus that the immunophenotypic selection and metabolic propensity of TRAMs are shaped by the local microenvironment 76-77. The proliferative activity of TRAMs gradually decreases with age 20, and this shift in immunophenotype may be the reason why elderly patients are more susceptible to airway diseases. In the BALF of healthy subjects, the ratio of M1-like to M2-like TRAMs was maintained between 1.04 and 2.2. This ratio in mice was 1.06 to 1.4, and in ALI/ARDS and IPF patients characterized by airway epithelial injury, the ratio of M1-like to M2-like TRAMs was seriously unbalanced (Mean_ALI/ARDS_ = 4.13, n = 114; Mean*_IPF_* = 3.74, n = 44). Although not strictly defined, M1-like TRAMs predominate in the ischemic and hypoxic environment of ALI/ARDS or IPF patients and therefore supply energy by means of anaerobic glycolysis, while in healthy controls, the dominant M2 is mainly the bioenergy style by OXPHOS.

Ca^2+^ release-activated Ca^2+^ (CRAC) channels and SOCE have been proven to be long-term Ca^2+^ signal transduction and immune function regulators in T-cell activation 78-79. STIMATE is the junction point of ERM-STIM1, and PM-ORAI1^29^ regulates the long-term maintenance of ER-plasma membrane junctions and the short-term physiological remodeling of the junctions 80. In the resting state of TRAMs, STIMATE binds to STIM1 located in the ERM. After ER [Ca^2+^] is exhausted, it quickly binds to PM-ORIA1 to open the Ca^2+^ channel. Under conditions of decreased STIMATE, the SOCE-dependent Ca^2+^ influx of TRAMs is inhibited, including transient (TG-induced) and long-acting (GM-CSF-induced) Ca^2+^ responsiveness. STIMATE^+^ ADEs interact with STIM1 in the ERM after being taken up by TRAMs so that TRAMs maintain instant and long-term high Ca^2+^ influx responsiveness. More efficient Ca^2+^ utilization promotes mitochondrial biogenesis activity and respiratory capacity in response to growth signals in TRAMs. The double-stranded circular mtDNA is approximately 16.5 kb and contains 37 genes encoding 13 subunits of ETC complexes I, III, IV and V, and correct mitochondrial biogenesis is dependent on approximately 1000 proteins encoded by the nuclear genome. Mitochondrial biogenesis is dependent on the activation of PGC-1α and the activity of CaN 48, which is a possible molecular mechanism by which STIMATE^+^ ADEs promote TRAMs to transform into OXOHOS. CaN inhibitors be widely used in clinical medicine to prevent allograft rejection and treat some autoimmune diseases, but they also often lead to renal interstitial fibrosis *81-82*. The increased STIM-ORAI bridge of STIMATE^+^ ADEs improves TRAMs Ca^2+^ responsiveness and long-term calcium mechanisms, thereby increasing the activity of cytoplasmic CaN, which controls the expression of PGC-1α in muscle and is involved in the increase in mitochondrial capacity 83. In addition to NRF, PGC-1α also interacts with other transcription factors and coactivates nuclear and mitochondrial gene transcription, such as PPAR and estrogen-related ERRα. The orphan nuclear receptors PPAR and ERRα target a common set of promoters involved in energy substrate uptake, ATP production and transport across mitochondrial membranes, and intracellular fuel sensing 84-85. Furthermore, DRP1 expression correlated with PGC-1α content and CaN activation in human skeletal muscle 86, and dephosphorylation of Drp1 by CaN induced its translocation to mitochondria, thereby triggering mitochondrial fission 87.

In conclusion, this study demonstrates the potential therapeutic effect of STIMATE^+^ ADEs in airway disease characterized by type II alveolar epithelial cell damage. STIMATE^+^ ADEs increase STIM-ORAI junctions and SOCE-dependent Ca^2+^ responsiveness and long-term calcium signaling, then enhance mitochondrial synthesis and OXPHOS capacity through PGC-1α-CaN signaling, thereby regulating metabolic reprogramming and immunophenotyping of TRAMs.

## Supplementary Material

Supplementary materials and methods, figures and tables.

## Abbreviations

AEC-Is: Type I alveolar epithelial cells
AEC-IIs: Type II alveolar epithelial cells
STIMATE: STIM-activating enhancer
STIMATE^+^ ADEs: STIMATE-positive AEC-IIs-derived exosomes
TRAMs: Tissue-resident alveolar macrophages
CaN: Calcineurin
rmSP-A: Recombinant surfactant proteins
ALI/ARDS: Acute lung injury/acute respiratory distress syndrome
IPF: Interstitial pulmonary fibrosis
AMs: Alveolar macrophages
TRAMs: Tissue-resident AMs
DAMPs: damage-associated molecular patterns
mtDNA: Mitochondrial DNA
PAMPs: Pathogen-associated molecular patterns
LPS: Lipopolysaccharide
TEM: Transmission electron microscopy
NTA: Nanoparticle Tracking analysis
GO: Gene Ontology
BALF: Bronchoalveolar lavage fluid
ECAR: Extracellular acidification rate
OCR: Oxygen consumption rate
OXPHOS: Oxidative phosphorylation
GR: Glycolytic reserve
FL-MOs: Fetal liver monocytes
BM-MOs: Bone marrow-derived monocytes
TIRF: Total internal reflection fluorescence
ERM: Endoplasmic reticulum membrane
SOCE: Store-operated Ca^2+^ entry
PM: Plasmic membrane
TG: Thapsigargin
SERCA: Sarco/endoplasmic reticulum Ca^2+^ ATPase
CCE: Cell cycle entry
ERC: Electron respiratory chain
Tfam: Mitochondrial transcription factor A
CaN: Calcineurin
CaMK: Ca^2+^-calmodulin-dependent kinase
DRP1: Dynamo-related protein
CsA: Cyclomycin A
CDE: Clathrin-dependent endocytosis
BLM: Bleomycin
iPSCs: Induced pluripotent stem cell
YS-Macs: Yolk sac-derived macrophages
FL-MOs: Fetal liver monocytes
CRAC: Ca^2+^ release-activated Ca^2+^
