# Supplementary material for: Exosomal STIMATE derived from type II alveolar epithelial cells controls metabolic reprogramming of tissue-resident alveolar macrophages
Source: Theranostics. 2023 Jan 22;13(3):991–1009. doi: 10.7150/thno.82552 (PMC9925314; doi:10.7150/thno.82552)
Supplement: Supplementary file 1 — Supplementary materials and methods, figures and tables. [file thnov13p0991s1.pdf]

Supplementary Materials and Methods

processing of human samples

100 ml of 0.9% sodium chloride aqueous solution was used to lavage the subjects' trachea and lower lung BALF through a bronchial microscope. After centrifugation at 1500 g for 5 min, the supernatant sample was used in an ELISA experiment to evaluate the content of $STIMATE^{+}$ exosomes in the lung. The obtained cell pellet was washed with 0.9% sodium chloride and centrifuged under the same conditions. The pellet was reselected in PBS containing 1% BSA and passed through a 20 μm cell sieve (Millipore, MA, USA) to obtain a single-cell suspension. The sample was loaded into the flow cytometer to detect the TRAMs in the alveoli. The ABL90 blood gas analyzer (Radiometer, Brønshøj, Denmark) was used to detect the oxygenation index PaO_2/FiO_2 in the whole peripheral blood of all subjects. The YH-830 medical ventilator (Yuwell, Shanghai, China) monitors the tidal volume, plateau pressure and PEEP of patients with PF to assess compliance with the respiratory system (Crs), Crs = tidal volume/(platform pressure-PEEP).

Animal experimental design

During aging, the TRAMs in the lungs of WT mice were gradually replaced by BMDMs. Therefore, all types of lung injury mice in the experiment were performed in young and middle-aged mice from newborn to 24 weeks of age. The mice were inhaled anesthetized by mixing 100 mg/kg ketamine (Sigma–Aldrich Corp. St., Louis, MO, USA) and 10 mg/kg xylazine (Sigma–Aldrich Corp. St., Louis, MO, USA) in a ratio of 1:1. 1 μl microinjector was used to intraperitoneal injection bleomycin (0.125 U/kg, Sigma–Aldrich, MO, USA). At the specified time point, the mice were euthanized by intraperitoneal injection of a 3-fold overdose of sodium pentobarbital (Aibei, Nanjing, China), and lung tissue, peripheral blood and BALF were collected for assessment of injury and fibrosis. For the pathological assessment of lung tissue injury, tracheal intubation was quickly performed after over injection of pentobarbital anesthesia, 1 ml 10% FPA was used for lung perfusion and internal fixation, the complete lung tissue was quickly removed and then external fixation was performed with 10% FPA for 48 h, Standard paraffin embedding and tissue section (4 μm) (Leica Biosystems, Wetzlar, Germany), H&E staining (Beyotime Shanghai, China), and then tissue damage and inflammation assessment. The pathological evaluation was independently performed by three experienced pathology clinicians, and the slices were scored as mild inflammation and injury (0-4), mild (5-8), moderate (9-12), or severe (13) -15). The FlexiVent pulmonary system (SCIREQ Inc. Montreal, Canada) was used to evaluate mouse respiratory system compliance (Crs) to observe the lung ventilation function of pulmonary fibrosis mice. For the detection of blood oxygen index, after an over injection of pentobarbital

anesthesia, 200 μl of blood was collected by intracardiac puncture, and the blood gas analyzer was used for detection within 30 min. After over injection of pentobarbital anesthesia, the mouse lungs were taken out, mixed with potassium hydroxide alkaline aqueous solution and boiled, adjusted to pH 6.5 with dilute hydrochloric acid, 4000 g, centrifuged for 10 min, and then used the hydroxyproline detection kit (Abcam, Cambridge, UK) to assess collagen deposition in the lungs of mice. For BALF STIMATE^{+} ADE content and M1-like/M2-like TRAM ratio detection, the trachea was washed 3 times with 1.5 ml sterile saline, and the samples were combined and centrifuged at 1500 g for 5 min. The supernatant was collected and used for ADE extraction and ELISA detection. The cell pellet was collected and passed through a 20 μM cell sieve (HyClone Laboratories, Logan, UT, USA) for flow cytometry.

Single-cell suspension preparation procedure

In short, mice were anesthetized by over injection of pentobarbital and perfused into the heart with 20 ml heparin (12500 U/ml, Aladdin, Shanghai, CHINA) in PBS. After opening the trachea, 1 ml of 1% low melting point agarose (Yeasen Biotechnology, Shanghai, CHINA) was injected at 42°C to fill the lungs. Carefully remove the entire lung tissue. Ophthalmic scissors divide the mouse lung into tissue fragments smaller than 1 mm3 and place them in 10 ml Digest the homogenate [(containing 2 mg/ml collagenase A (Roche, Mannheim, GER) and 1XDNase I (Sigma–Aldrich, MO, USA)] in Dulbecco's modified Eagle medium (DMEM, HyClone, UT, USA) for 30 min, Shake at 37°C. Two milliliters of fetal bovine serum (HyClone, UT, USA) was used to terminate the digestion. A microsyringe was used to pass the homogenate through a 70-, 40- and 20-μm nylon mesh filter membrane (Millipore, MA, USA). After centrifugation at 2000 g and 4°C for 5 min, the cell pellet was resuspended in PBS containing 1% BSA.

Confocal laser scanning microscopy (CLSM) and total internal reflection fluorescence microscopy (TIRF)

TRAMs or AEC-IIs were previously seeded on 35 mm glass-bottom culture dishes (MatTek, MA, USA) at very low density and grown overnight at 37 °C and 5% CO_2 using 10 mM HEPES, 100 μg ml^{-1} DMEM (HyClone, UT, USA) with penicillin, 100 μg ml^{-1} streptomycin and 10% heat-inactivated fetal bovine serum (Exosome free, Gibco, CA, USA) to clean nonadherent cells. After adding STIMATE^{+}-ADEs or using thapsigargin (Abcam, MA, USA)) to induce storage depletion or rmGM-CSF (Gibco, CA, USA) stimulation, CLSM and TIRF imaging were performed according to the experimental requirements. TIRF images were obtained on a NIKON Eclipse Ti-E microscope (Tokyo, Japan), and CLSM were

obtained on a Leica STELLARIS DIVE confocal system (Wetzlar, Germany). A 488 nm laser was used to excite GFP, a 561 nm laser was used to excite mCherry, and a 400 nm laser was used to excite DAPI or the excitation wavelength indicated in the instructions of the corresponding fluorescently labeled antibody. For the observation of AEC-IIs, exosome release and the thapsigargin-excited store-operated Ca^{2+} entry (SOCE) experiment of TRAMs, 100 nm fluorescent beads (TetraSpecK microspheres, Life TeChinaologies, MD, USA) were deposited on a cover glass and imaged as a mark for later alignment. Image analysis was performed using NIS-Elements software (Nikon, Tokyo, Japan) or ImageJ (National Institutes of Health, MD, USA).

Ca^{2+} measurement and imaging in TRAMs

The intracellular Ca^{2+} measurement was performed with Fura-2 AM (MedChemExpress, NJ, CHINA) dye, and the intracellular Ca^{2+} concentration was measured by the ratio of the fluorescence intensity of the alternating excitation wavelengths of 340 nm and 380 nm, as described in our previous study[28] . In short, 24 h before imaging, WT or STIMATE$^{-/-}$ TRAMs were seeded on a 35 mm glass-bottom culture dish (MatTek, MA, USA) at a medium density. After preincubating TRAMs with different genetic backgrounds for 12 h, Fura-2 AM was loaded. Before imaging, TRAMs were stored in an imaging solution containing 1 mM CaCl2 and 2 µM Fura-2 AM for 60 min. After that, the cells were cultured in Ca^{2+}-free standard DMEM (HyClone, UT, USA). For the determination of long-acting Ca^{2+} accumulation induced by GM-CSF, 10% exosome-free fetal bovine serum (Gibco, CA, USA) was used. Add 1 µM thapsigargin, 100 ng/ml rm GM-CSF and 1.2 mM $CaCl_2$ solution according to the time point of the experimental procedure. A single-frequency microscope filter set was used to alternately excite cells at 340 nm and 380 nm, and 409 nm single-frequency and 510/84 nm dichroic filters were used to monitor the emission wavelength. A Nikon inverted microscope (400 x oil immersion objective lens, NA = 1.4) (Tokyo, Japan) was used to enlarge the image, and an EMCCD camera was used to capture the image (EM gain was set to the linear dynamic range, the exposure time was 50 milliseconds, and images were acquired at 10-second intervals). For the thapsigargin-excited SOCE experiment, the area under the curve (ROC) with a fluorescence intensity ratio of 340 nm/380 nm between 200 and 400 s was taken as the calcium storage in the endoplasmic reticulum and the Ca^{2+} influx peak at 340 nm/380 nm after adding $CaCl_2$ solution response capacity for SOCE. For the measurement of long-acting Ca^{2+} accumulation induced by GM-CSF, the area under the curve (ROC) with a fluorescence intensity ratio of 340 nm/380 nm from 0 to 250 s was taken as

the total accumulation value of the intracellular calcium response. All experiments were performed at room temperature.

Processing of extracellular vesicles

In short, the obtained human BALF, mouse BALF or cell culture medium was centrifuged at 300 g for 5 min to remove exfoliated inflammatory cells or dead cells. Then, the supernatant was collected and centrifuged at 1,200 g for 10 min to remove cell debris to obtain a pretreated sample. Load 25 ml of pretreated sample supernatant to the top of an Amicon® Ultra-50 45 kDa Centrifugal Filter Unit (Merck, MA, USA), 75 min at 45000 g at 4 °C. Next, 3.5 ml of the PBS-eluted was transferred to a new Amicon® Ultra-14 35 kDa Centrifugal Filter Unit, diluted with 60 ml of PBS and centrifuged for 70 min at 45000 g at 4 °C. The purification and identification of exosomes require considerable time and the loss of many samples. For this reason, ELISA was used to quickly detect the relative concentration of $CD63^{+}$ exosomes or $STIMATE^{+}$ ADE in BALF or in cell culture media. To detect CD63 or STIMATE on exosomes, each well of an ELISA-embedded plate (96-well) (Abcam, MA, USA) was coated with 0.25 μg of anti-CD63 or STIMATE monoclonal antibody overnight at 4 °C. Two hundred microliters of blocking buffer (Pierce, IL, USA) was incubated for 1 h at room temperature to block free binding sites. Add 100 μl of the above-pretreated sample to each ELISA cell. After incubating overnight at 4 °C, a biotin-labeled monoclonal CD63 or STIMATE antibody was added and incubated at room temperature for 1 h. Then, 100 μl horseradish peroxidase-conjugated streptavidin (B & D Biosciences, NJ, USA) was added and incubated at room temperature for 1 h. Tetramethylbenzidine (Pierce, IL, USA) was used as a color developer, and 0.5 N H_2SO_4 was used to stop the reaction. A BioTek microplate reader (Vermont, USA) was used to read the absorbance value of the ELISA plate at an emission wavelength of 450 nm. Recombinant human CD63 or STIMATE protein (Gibco, CA, USA) was used to generate a standard curve. Recombinant P-selectin protein (Gibco, CA, USA) was used as a negative control to verify the specificity of the test.

OCR and ECAR analysis

A Seahorse XFe96 Cell Metabolic Respiratory Dynamics Analyzer (Seahorse Bioscience, Inc., North Billerica, MA) was used to measure the oxygen consumption rate (OCR) and extracellular acidification rate (ECAR) of TRAMs. The cells were cultured in a 96-well plate containing 10 mM glucose, 1 mM GlutaMAX (Sigma–Aldrich, MO, USA) and 1 mM sodium pyruvate in XF medium (Agilent Technologies, CA, USA). The following drugs were added according to the program settings: ATP

synthase inhibitor oligomycin (1 μM); proton carbonyl cyanide-4-(trifluoromethoxy)phenylhydrazone (FCCP) (0.75 μM) uncoupling mitochondria; mitochondrial complex I inhibitor rotenone (100 nM) + mitochondrial complex III inhibitor antimycin A (1 μM) (all inhibitors were obtained from Agilent Technologies, CA, USA). The basal oxygen consumption rate (OCR) was calculated by subtracting the OCR after rotenone and antimycin A treatment from the OCR before oligomycin treatment. The maximum OCR was calculated by subtracting the OCR after rotenone and antimycin A treatment from the OCR measured after adding FCCP. The measurements before any reagents are introduced are baseline OCR and ECAR. Oligomycin is a compound V inhibitor that blocks the production of ATP, and the measurement after addition is the OCR and ECAR related to ATP production. Adding FCCP to destroy the inner mitochondrial membrane gradient was measured as the maximum OCR and ECAR of the cell. Antimicrycin A and rotenone were added to turn off ETC complex activity to measure the OCR and ECAR from nonmitochondria.

Aerosol inhalation quantify

The total protein concentration of exosomes isolated from the BALF of healthy mice was 11.2 ± 4.17 μg/ml, as quantified by the BCA method. The concentration of STIMATE protein in exosomes isolated from the BALF of healthy mice was 2.2 ± 1.2 ng/ml, as detected by EV-based ELISA. The number of BAFL exosomes in healthy mice was $1.23\times10^{8} \pm 3.3\times10^{6}$/ml, as assessed by NTA. We obtained 1-1.5 ml BALF from each mouse by lavage with PBS. Considering that the number of mouse lung particles will be diluted with PBS, it needs to reach 10^{9} to 10^{10} particles; that is, 27-270 ng STIMATE^{+}-ADE per mouse can restore physiological STIMATE^{+}-ADE concentrations in injured mice. A total of 4×10^{6} primary AEC-IIs (primary cultures from 6 mice) secreted approximately 2-3 ng STIMATE^{+}ADE 24 h after mtDNA transfection, thus severely limiting the therapeutic application of STIMATE. MLE-12 cells (2^107) can obtain approximately 2-3 ng of STIMATE^{+}-ADE within 24 h of mtDNA transfection, whereas rmSP-A/mtDNA agarose particle induction (low temperature and hypoxia) can harvest approximately 30-75 ng. Therefore, for each mouse inhalation treatment, 8×10^{7} MLE-12 cells (approximately 3 filled 75 ml cell culture flasks) were cocultured with 8 μM rmSP-A/mtDNA agarose pellets for 24 h.

Glucose utilization and glucose uptake measurement

The fluorescent glucose analog 2-NBDG (Abcam, MA, USA) was used to indirectly analyze the glucose uptake ability of TRAMs, according to the product specification. In short, TRAMs were cultured in

glucose-free RPMI 1640 medium 6 h before the experiment, and 100 ng/ml rm GM-CSF was added to induce proliferation after preincubating TRAMs with STIMATE^{+} ADEs of different genetic backgrounds for 12 h. After 12 h, the specified concentration of 2-NBDG was added and incubated for 10 minutes. The cell culture supernatant was removed, and the cells were washed with cell-based assay buffer. At this time, propidium iodide was added to exclude dead cells if analyzed by flow cytometry. Cell detection buffer and immediately performed flow cytometry analysis. Flow cytometry and FlowJo software were used to detect the fluorescence intensity of TRAMs at wavelengths of 485/535 nm.

Flow Cytometry

For the cell ratio of TRAMs and BMDMs and the detection of AEC-II, the pretreated BALF cell pellet was resuspended in PBS containing 0.1% BSA. Anti-FcγRII/FcγRIII (Alomone Laboratories, Jerusalem, Israel) was used for blocking for 5 min. Primary antibodies coupled with different fluorescence wavelengths were added according to the predetermined antibody dilution ratio and incubated for 30 min in the dark. For the immunocoupling of intracellular factors, Triton X-100 (Sigma–Aldrich, MO, USA) was used; after permeabilization, the primary antibody was added according to the product instructions. The stained cells were analyzed using a FACSVerse flow cytometer (BD Biosciences, NJ, USA) and FlowJo software (Version 10.7; TreeStar, OR, USA). The cell sorting strategy is shown in FIG. S8, and the commercialized antibody information is shown in Supplementary Table S1.

Western blot

SDS–PAGE was performed on an equal amount of lysate under each condition, transferred to a nitrocellulose membrane, blocked with low-fat milk, and incubated with the specific WB primary antibody mixture overnight. The protein was visualized using HPR-conjugated anti-mouse secondary antibody (Sigma–Aldrich, MO, USA) and chemiluminescent ECL reagent (Thermo Fisher Scientific, MA, USA), and the optical density was quantified using ImageJ (National Institutes of Health, MD, USA).

Real-time fluorescent quantitative PCR

TRIzol (Invitrogen, CA, USA) was used to isolate total RNA, and an iScript cDNA synthesis kit (Bio-Rad, CA, USA) was used to synthesize cDNA. Maxima SYBR Green qPCR Master Mix (Thermo Fisher, MA, USA) and gene-specific primers for quantitative real-time PCR. The relative abundance of transcripts was normalized to the expression of housekeeping genes using the 2-ΔCT method. The relative expression value was calculated by normalizing gene expression to the average value of untreated WT TRAMs.

Supplementary Figure.

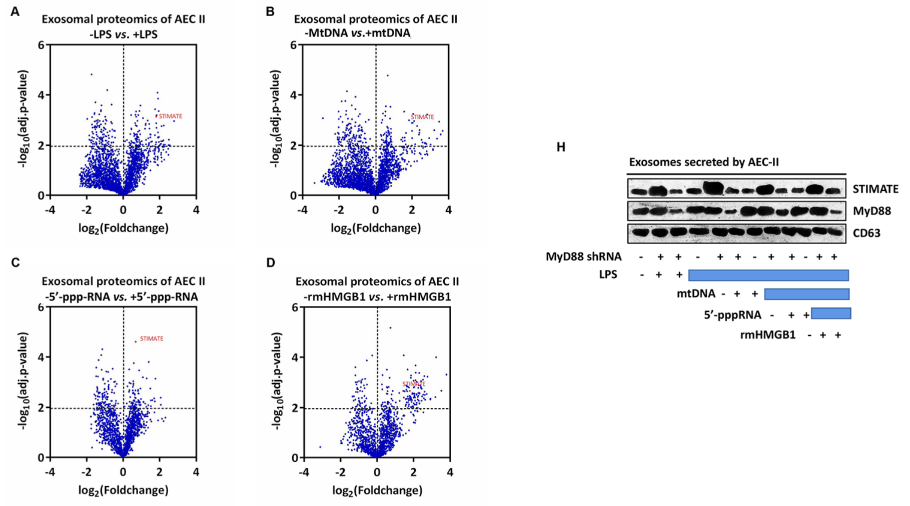

E

Biological process	-Log10[PValue]	Count
Cell-cell adhesion	9.48E+00	14
Viral transcription	8.63E+00	10
Nuclear-transcribed mRNA catabolic process	8.40E+00	10
SRP-dependent cotranslational protein targeting to membrane	7.92E+00	9
Translational initiation	6.63E+00	9
rRNA processing	5.18E+00	9
Viral process	5.00E+00	10
Translation	4.65E+00	9
Extracellular matrix organization	4.46E+00	8
Movement of cell or subcellular component	4.31E+00	6
Antigen presentation via MHC class I, TAP-dependent	3.68E+00	5
Cell adhesion	3.57E+00	10
Positive regulation of vesicle fusion	3.36E+00	3
Ephrin receptor signaling pathway	3.16E+00	5
Wnt signaling pathway, planar cell polarity pathway	3.05E+00	5
Proteolysis involved in cellular protein catabolic process	2.843213278	4
Regulation of cellular amino acid metabolic process	2.766909918	4
Cell adhesion mediated by integrin	2.667433026	3
Positive regulation of receptor-mediated endocytosis	2.460850766	3
NIK/NF-kappaB signaling	2.446651802	4

F

Cellular component	-Log10[PValue]	Count
Extracellular exosome	28.76037043	58
Focal adhesion	19.46552541	24
Cell-cell adherens junction	12.28794288	17
Membrane	12.07573375	36
Vesicle	9.708311801	11
Melanosome	7.897159473	9
Cytosol	7.704615586	37
Myelin sheath	6.511745847	9
Cytoplasm	6.370686389	45
Cytosolic large ribosomal subunit	6.315525882	7
Cell surface	6.267153217	14
Intracellular ribonucleoprotein complex	5.689158863	8
Membrane raft	5.515402274	9
Ribosome	5.115766467	8
Plasma membrane	4.377940394	35
Blood microparticle	4.289569478	7
Extracellular vesicle	4.188868863	5
Proteasome core complex	4.006253747	4
Basolateral plasma membrane	3.88382224	7
Cell cortex	3.709503362	6

G

Molecular Function	-Log10[PValue]	Count
Cadherin binding involved in cell-cell adhesion	12.69240698	17
Protein binding	10.1193468	68
Poly(A) RNA binding	7.714182151	22
Structural constituent of ribosome	5.118591652	9
GTP binding	4.213336041	10
GTPase activity	4.023134165	8
Threonine-type endopeptidase activity	3.941610044	4
Integrin binding	3.931278189	6
RNA binding	3.736791565	11
Structural constituent of cytoskeleton	2.795157412	5
Calcium ion binding	1.788795465	9
Phospholipase A2 inhibitor activity	1.741781696	2
Actin filament binding	1.648494702	4
Neuregulin binding	1.645848367	2
Calcium-dependent protein binding	1.540683744	3
Calcium-dependent phospholipid binding	1.540683744	3
Double-stranded RNA binding	1.500296764	3
Cell adhesion molecule binding	1.48731919	3
Glycoprotein binding	1.449733314	3
Nitric-oxide synthase regulator activity	1.444654243	2

Figure S1. Characterization of exosomal protein cargo.

(A-D) Volcano map of differentially existing protein cargo in exosomes released by mouse AEC-IIs before and after stimulation by LPS and rmHMGB1 or transfection by mtDNA and 5'-pppRNA. Fold change>1.5

or <0.5 and p value<0.01 were considered statistically significant, and upregulated proteins in the four panels were used for overlap analysis in the Venn diagram.

(E-F) GO analysis of the overlap of differentially existing protein cargos in exosomes, including biological processes, cellular components and molecular functions.

(G) Representative Western blotting image of STIMATE protein expression in exosomes released by mouse AEC-IIs and myD88 shRNA-transfected AEC-IIs after stimulation with LPS and rmHMGB1 or transfection with mtDNA and 5'-pppRNA. n=3. CD63 and represent Exo-specific markers.

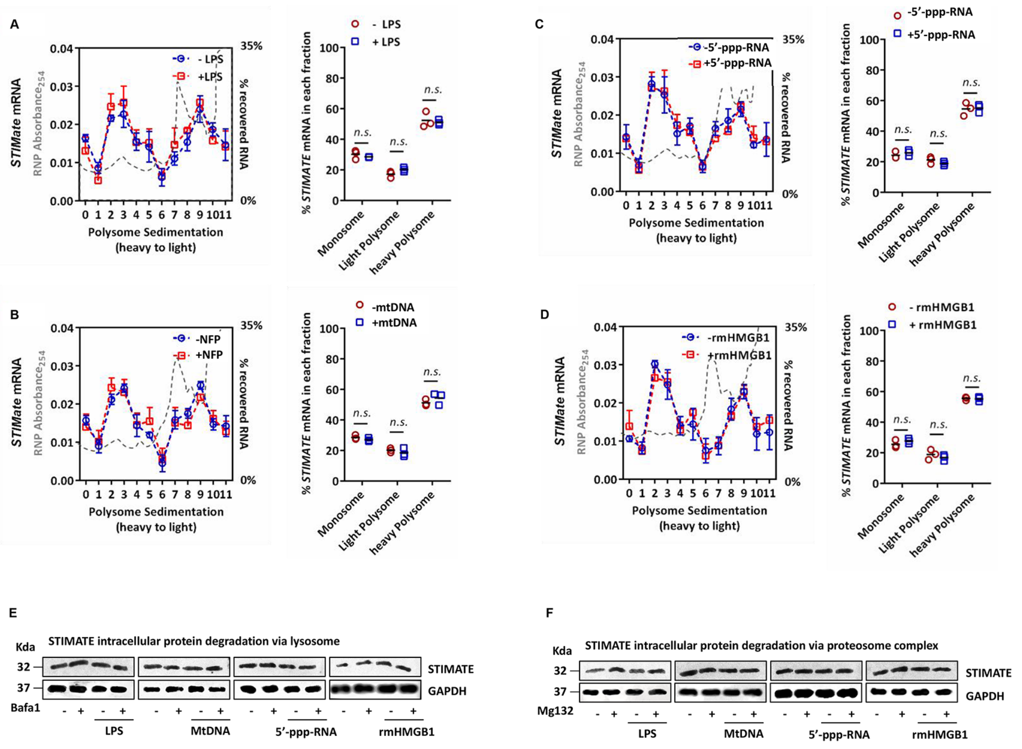

Figure S2. The effect of differences in protein translation and degradation on STIMATE expression in AEC-IIs.

(A-D) Polysome profiles in LPS-, rmHMGB1-, mtDNA-, 5'-pppRNA-induced mouse AEC-IIs and the composition of STIMATE mRNA across the monosome, low-polysome, and heavy-polysome fractions in AEC-IIs. Separation of polysomes by sucrose density gradient centrifugation. The dashed gray line is the absorbance of ribosomes at 254 nm. The abscissa represents relatively heavy polyribosomes from left to right, single ribosomes (80S), ribosomal subunits (60S), 40S and mRNP single molecules (50% to 15% sucrose). The colored dotted line is the gene mRNA change detected by RT–PCR.

(E) A representative western blot of STIMATE in mouse AEC-IIs induced by LPS-, rmHMGB1-, mtDNA-, or 5'-pppRNA in the presence or absence of the lysosome inhibitor BafA is shown.

(F) A representative western blot of STIMATE was performed in mouse AEC-IIs induced by LPS, rmHMGB1, mtDNA, or 5'-pppRNA in the presence or absence of the proteasome inhibitor MG132.

Data are shown as the mean±SD, *p<0.05 and **p<0.01 by paired Student's t test compared to untreated AEC-IIs. The proteomics of exosomes comes from 2 independent experiments, and the polysome and western blot data come from 3 independent experiments.

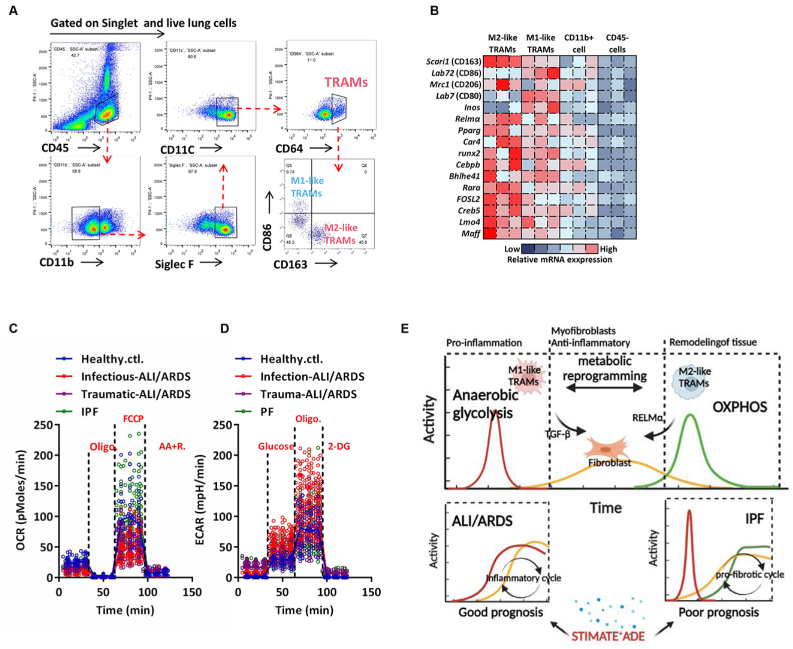

Figure S3. Immune characteristics of M1-like TRAMs and M2-like TRAMs.

(A) Flow cytometry sorting strategy for the collection of M1-like ($CD86^+$) and M2-like ($CD163^+$) TRAMs ($CD45^+CD11B^-$Siglec $F^+CD11D^+CD64^+$) from patient BALF.

(B) Heatmap of AM-specific transcription factor gene expression from the BALF of two ALI/ARDS patients and one IPF patient in M1-like and M2-like TRAMs compared to CD45-nonimmune cells and $CD11B^+$ dendritic cells, showing log normalized values from minimum (blue) to maximum (red).

(C) Seahorse TRAM mitochondrial respiration monitoring in BALF of healthy subjects, infectious & traumatic ALI/ARDS and interstitial PF patients. OCR at baseline and after sequential treatment with oligomycin (Oligo), carbonyl cyanide 4-(trifluoromethoxy) phenylhydrazone (FCCP) and antimycin A & rotenone (AA+R).

(D) Seahorse TRAMs glycolysis capacity test in BALF of healthy subjects and patients. ECAR at baseline and after sequential treatment with oligomycin (Oligo) and 2-deoxy-D-glucose (2-Dg).

(E) Schematic diagram of lung homeostasis: Immunophenotypic selection and metabolic predisposition of TRAMs in early-stage inflammation (red curve), intermediate proliferation (yellow curve), and late-stage resolution/scar formation (green curve) aiming for lung tissue homeostasis.

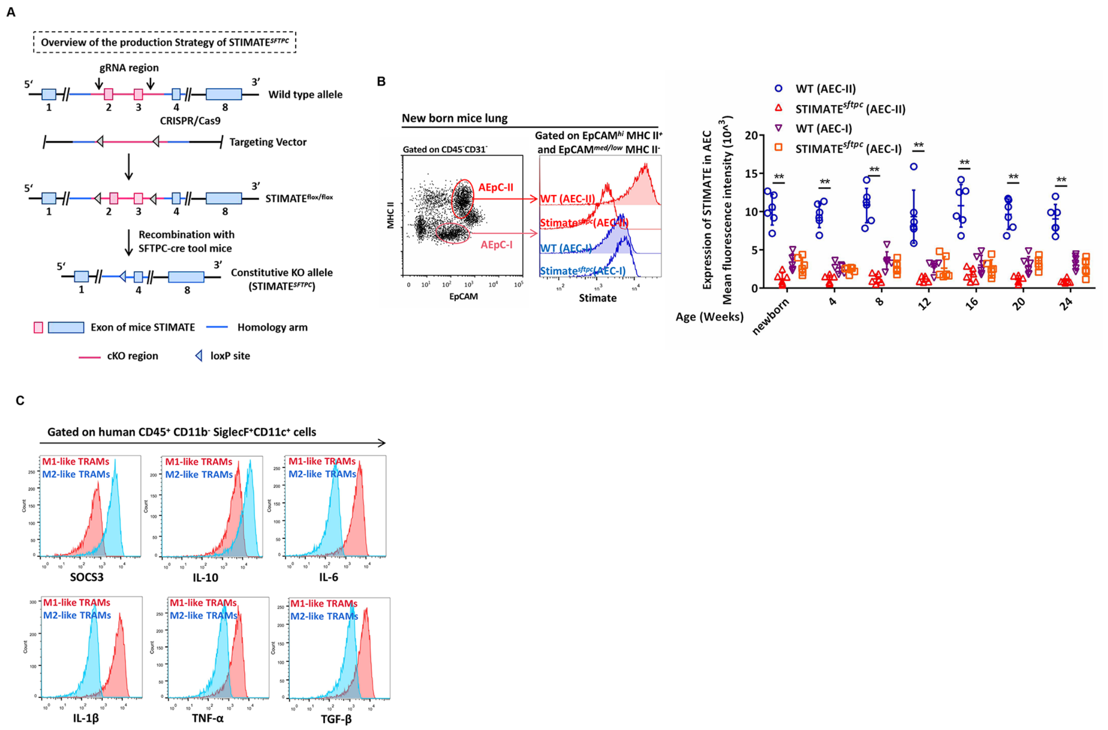

Figure S4. STIMATE*sftpc*-deficient mice.

(A) The production and reproductive pattern of STIMATE*sftpc*-deficient mice.

(B)Flow cytometry analysis of STIMATE expression in AEC-I and AEC-IIs of STIMATE*sftpc* mice, the right plot data based on STIMATE knockout efficiency in AEC-I and AEC-IIs in multiple age groups, n = 6.

(C) Flow cytometric analysis showing the expression of the cytokines SOCS3, IL-10, IL-6, IL-1β, TNF-α and TGF-β on M1-like human TRAMs (red) and M2-like human TRAMs (blue).

Data are shown as the mean±SD, $*p < 0.05$ and $**p < 0.01$ by paired Student's t test compared to untreated AEC-IIs. The proteomics of exosomes comes from 2 independent experiments, and the polysome and western blot data come from 3 independent experiments.

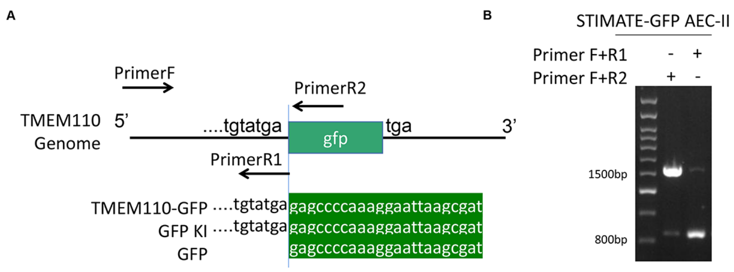

Figure S5. Construction of STIMATE-gfp AEC-IIs.

(A) Diagram of the inserted location of STIMATE-GFP knock-in (KI) mouse AEC-IIs and sequencing of the genome. (B) PCR analysis of STIMATE-GFP knock-in (KI) AEC-IIs.

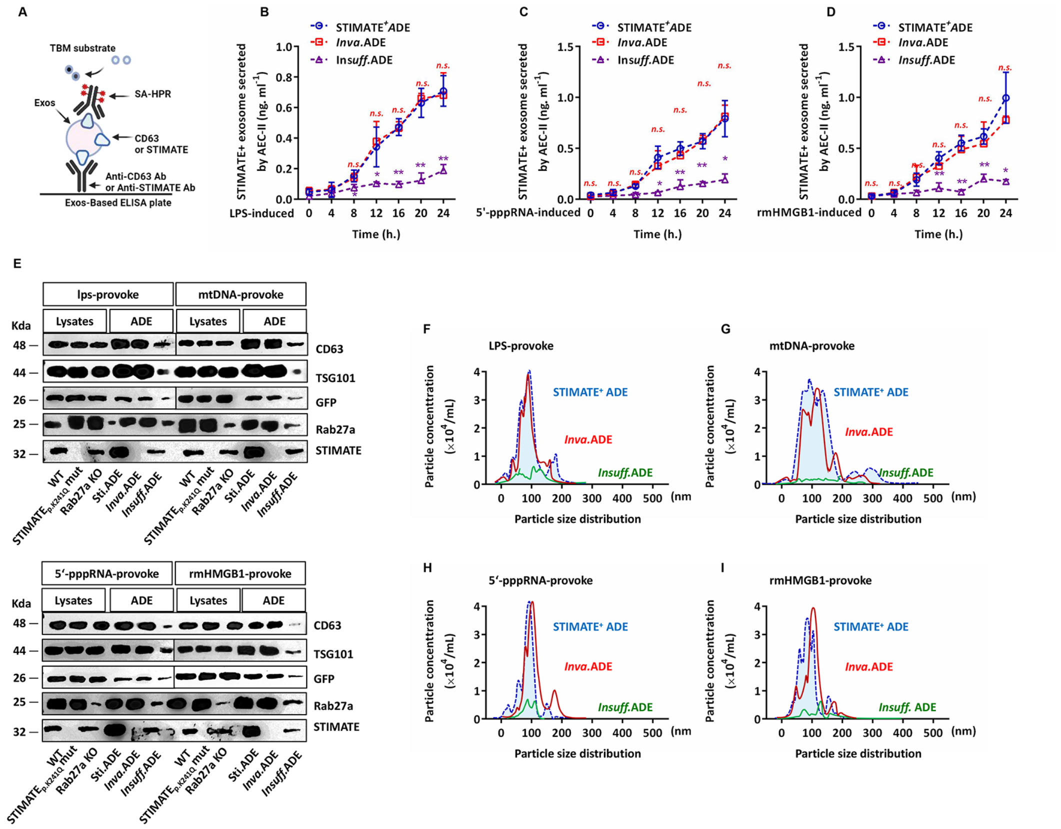

Figure S6. Characteristics of ADE released by different genetic backgrounds of AEC-IIs and different provocative AEC-IIs.

(A) Schematic diagram of exosomes-based ELISA for detecting exosomal CD63 or STIMATE. For more information, please refer to the methods.

(B-D) exosomes-based ELISA detects STIMATE^{+}ADE, *Inva.*ADE and *Insuff.*ADE released by GFP-tagged STIMATE WT, STIMATE$^{P.\ K241Q}$ and Rab27a KO mouse AEC-IIs after transfection with 5'-pppRNA and stimulation with LPS and rmHMGB1 after 24 h, respectively. Draw according to the reorganized STIMATE standard curve.

(E) Representative Western blotting image of STIMATE and Rab27a protein expression in GFP-tagged STIMATE WT and STIMATE$^{P\ cells.\ K241Q}$ and Rab27a KO AEC-IIs lysates and exosomes after transfection with 5'-pppRNA and stimulation with LPS and rmHMGB1 after 24 h, respectively, n=3. CD63 and Tsg101 represent Exo-specific markers.

(F-I) The NanoSight nanoparticle tracking system characterizes the release of ultrafiltration centrifugation exosomes from GFP-tagged WT, STIMATE$^{P.\ K241Q}$ and Rab27a KO mouse AEC-IIs stimulated by LPS & rmHMGB1 and transfected by mtDNA & 5'-pppRNA, respectively.

Data are shown as the mean±SD, $*p < 0.05$ and $**p < 0.01$ by analysis of variance (ANOVA) followed by a post hoc test compared to resting TRAMs. All data were derived from 3 independent experiments.

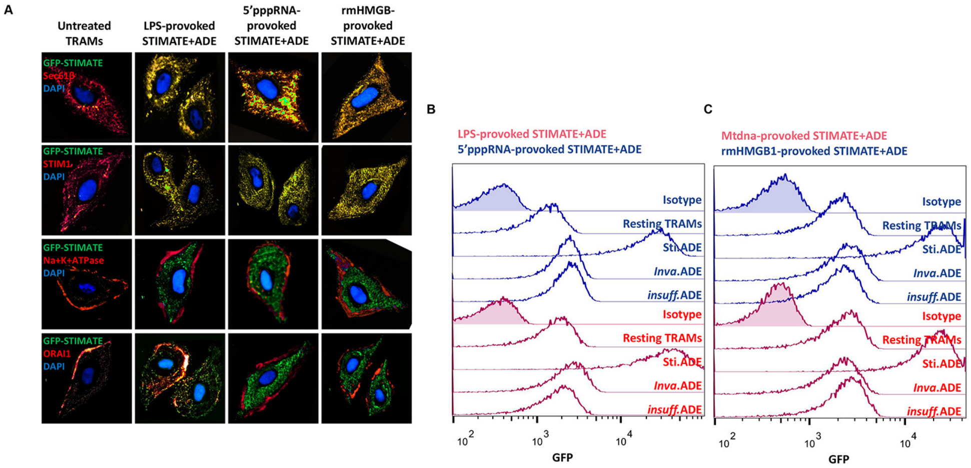

Figure S7. Uptake of ADE by mouse TRAMs released by different genetic backgrounds of AEC-IIs and different provocative AEC-IIs.

(A) Representative CLSM of mouse TRAMs after 12 h of cocultivation with lps, 5'-pppRNA and rmHMGB1 provoked STIMATE^{+}ADE. The images from top to bottom sequentially show the merged GFP-STIMATE with anti-Sec61β, anti-STIM1, anti-Na^{+}Ka^{+} ATPase and anti-ORAI1. Scale bar: 2 μm.

(B-C) The fluorescence intensity of GFP in mouse TRAMs obtained from STIMATE^{+}ADE that were released from different genetic backgrounds AEC-IIs and different provocative AEC-IIs was measured by flow cytometry.

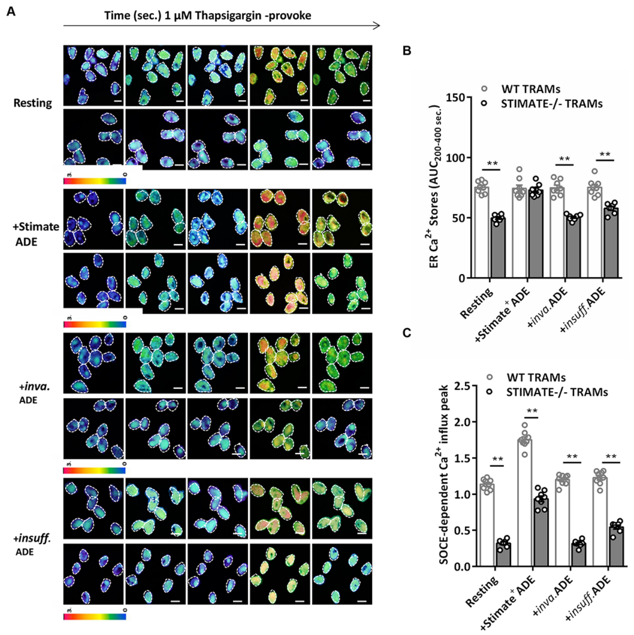

Figure S8. Ca^{2+}storage capacity assay

(A) The representative image shows the fluorescence image of GFP-positive mouse TRAMs (defined by the dotted circle) loaded with Fura-2 AM in TRAMs at representative time points with $STIMATE^{+}$-ADE, *Inva.*ADE and *Insuff.*ADE before 1 μM TG induction.

(B and C) The graph shows the quantification of ER Ca^{2+} storage capacity and SOCE Ca^{2+} influx peak. Error bars denote s.e.m. for n = 8 cells pooled across 3 independent experiments.

Data are shown as the mean±SD, *p<0.05 and **p<0.01 by paired Student's t test compared to untreated AEC-IIs. The proteomics of exosomes comes from 2 independent experiments, and the polysome and western blot data come from 3 independent experiments.

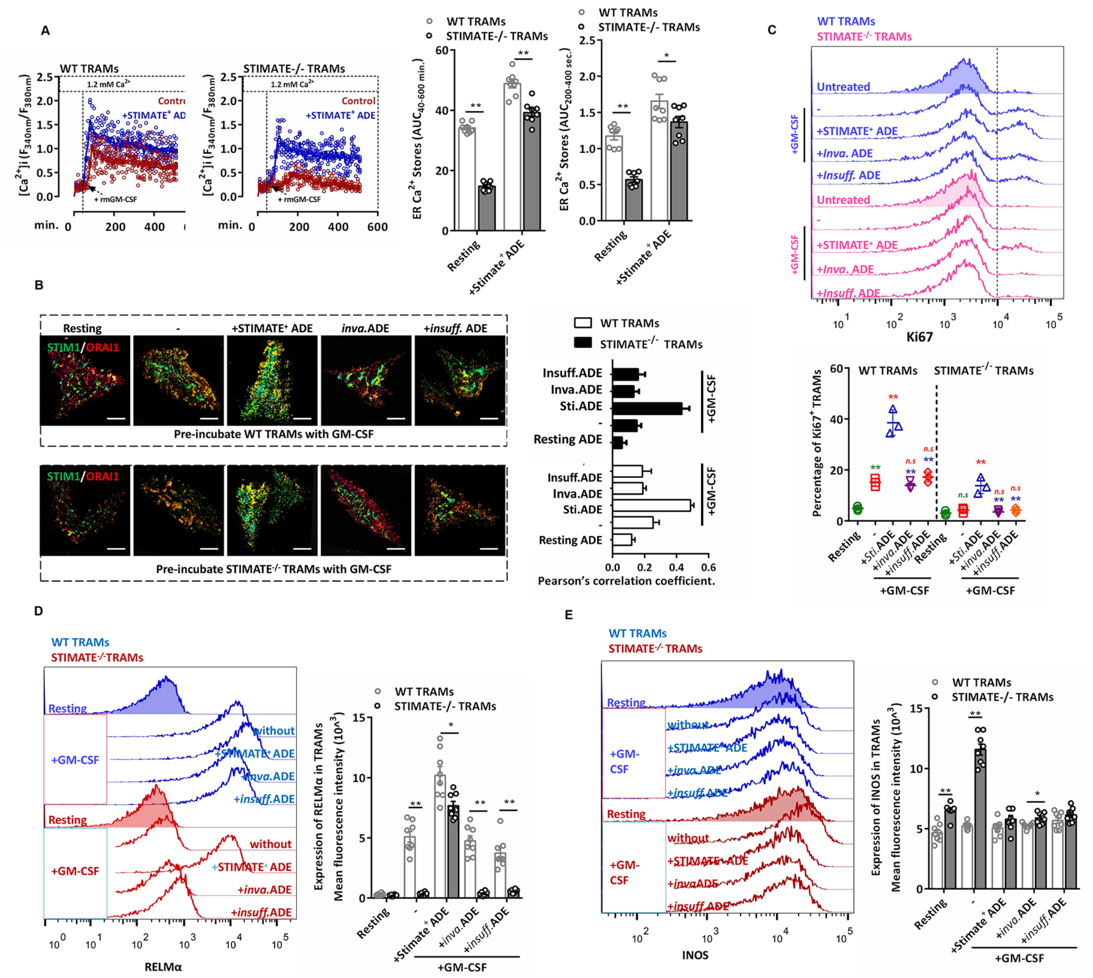

Figure S9. Effects of STIMATE$^+$ADE on GM-CSF-induced long-term Ca^{2+} influx and CCE in TRAMs.

(A) Representative images of Ca^{2+} influx in WT or STIMATE$^{-/-}$ mouse TRAMs preincubated with STIMATE$^+$ADE, *Inva.*ADE and *Insuff.*ADE before rmGM-CSF induction. The left figure shows the ratio of excitation wavelengths of Fura-2 AM at 340 nm and 380 nm. The right panel shows the quantification of ER Ca^{2+} storage capacity and SOCE Ca^{2+} influx peak. Error bars denote s.e.m. for n = 8 cells pooled across 3 independent experiments. Scale bar, 30 µm.

(B) Representative TIRF image of STIM1-GFP tag/ORAI1-mCherry tag cotransfected WT or STIMATE$^{-/-}$ mouse TRAMs stimulated with rmGM-CSF for 8 h after cocultivation with STIMATE$^+$ADE, *Inva.*ADE and *Insuff.*ADE for 12 h, sequentially. The bar graph on the right shows the Pearson's correlation coefficient of the fused fluorescent spots. Scale bar, 2 µm.

(C) Flow cytometry analysis of the expression of the cell cycle entry checkpoint Ki67 in WT or STIMATE$^{-/-}$ mouse TRAMs induced by GM-CSF after cocultivation with STIMATE$^+$ADE, *Inva.*ADE and *Insuff.*ADE for 12 h.

(D and E) Flow cytometry analysis of RELMα and INOS expression in WT or STIMATE$^{-/-}$ mouse TRAMs induced by GM-CSF after cocultivation with STIMATE$^+$-ADE, *Inva.*ADE and *Insuff.*ADE for 12 h.

Data are shown as the mean±SD, *p < 0.05, and**p < 0.01 by analysis of variance (ANOVA) followed by a post hoc test compared to resting TRAMs. All data were derived from 3 independent experiments.

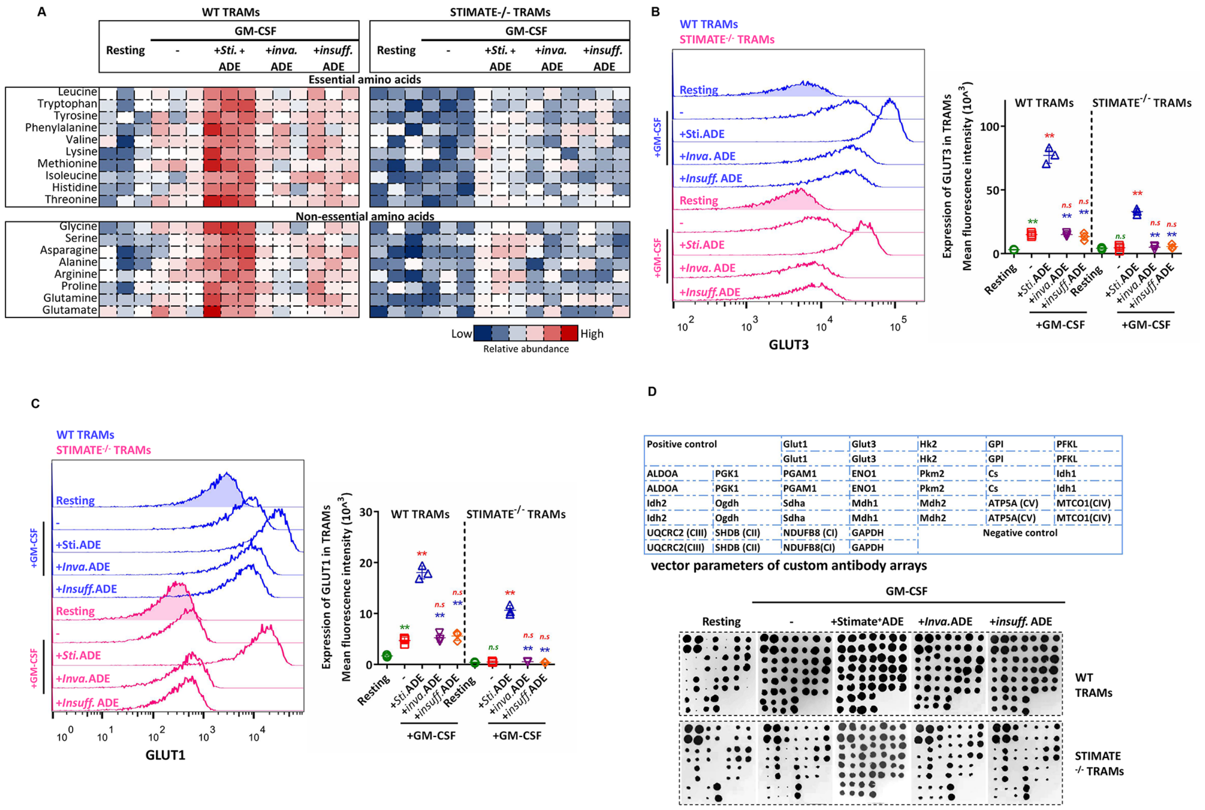

Figure S10. STIMATE^{+}ADE affects amino acid metabolism and glucose utilization in TRAMs

(A) The abundance of polar metabolites of essential and nonessential amino acids in WT or STIMATE$^{-/-}$ mouse TRAMs induced by GM-CSF after cocultivation with STIMATE^{+}ADE, *Inva.*ADE and *Insuff.*ADE for 12 h. Heatmap showing log normalized values from minimum (blue) to maximum (red).

(B and C) Flow cytometry analysis of the expression of the glucose transporters GLUT1 and GLUT3 in WT or STIMATE$^{-/-}$ mouse TRAMs induced by GM-CSF after cocultivation with STIMATE^{+}ADE, *Inva.*ADE and *Insuff.*ADE for 12 h.

(D) The upper panel is mouse antibody pair List vector parameters of custom antibody arrays. The lower panel is the inverse phase image of the fluorescence luminescence of antibody arrays.

Data are shown as the mean±SD, *p < 0.05, and **p < 0.01 by analysis of variance (ANOVA) followed by a post hoc test compared to resting TRAMs. All data were derived from 3 independent experiments.

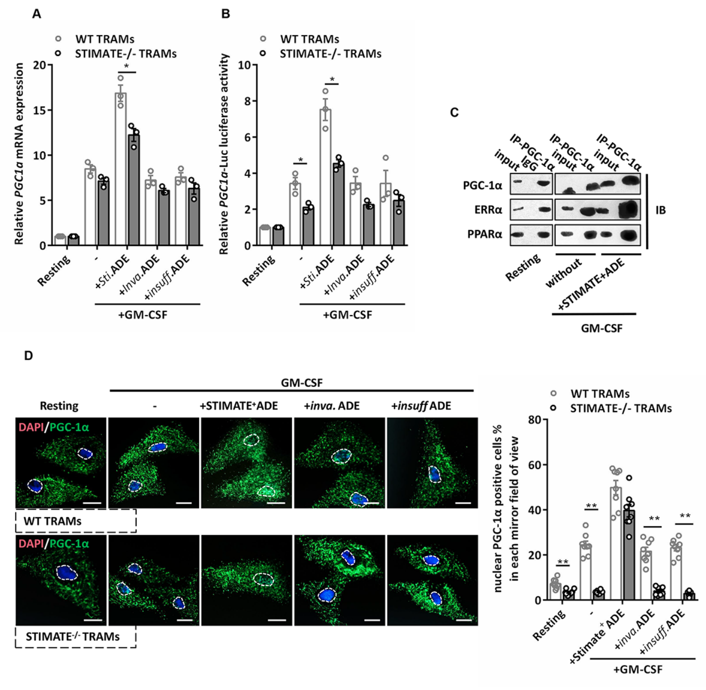

Figure S11. Expression and activation of PGC-1α.

(A) PGC-1α mRNA expression in WT or STIMATE$^{-/-}$ mouse TRAMs preincubated with STIMATE^{+}-ADE, *Inva.*ADE and *Insuff.*ADE for 12 h before rmGM-CSF induction.

(B) Fluorescence intensity of the transfected PGC-1α-luc luciferase reporter gene in WT or STIMATE$^{-/-}$ mouse TRAMs preincubated with STIMATE^{+} ADE, *Inva.*ADE and *Insuff.*ADE for 12 h before rmGM-CSF induction.

(C) Representative images of coimmunoprecipitation analysis of the interaction of PGC-1α with ERRα and PPARα in WT or STIMATE$^{-/-}$ mouse TRAMs preincubated with STIMATE^{+}-ADE for 12 h before rmGM-CSF induction.

(D) Representative immunofluorescence image of nuclear DAPI and PGC-1α in WT or STIMATE$^{-/-}$ mouse TRAMs preincubated with STIMATE^{+}-ADE, *Inva.*ADE and *Insuff.*ADE for 12 h before rmGM-CSF induction. The bar graph on the right depicts the quantification of the indicated number of cells. Scale bar, 10 μm.

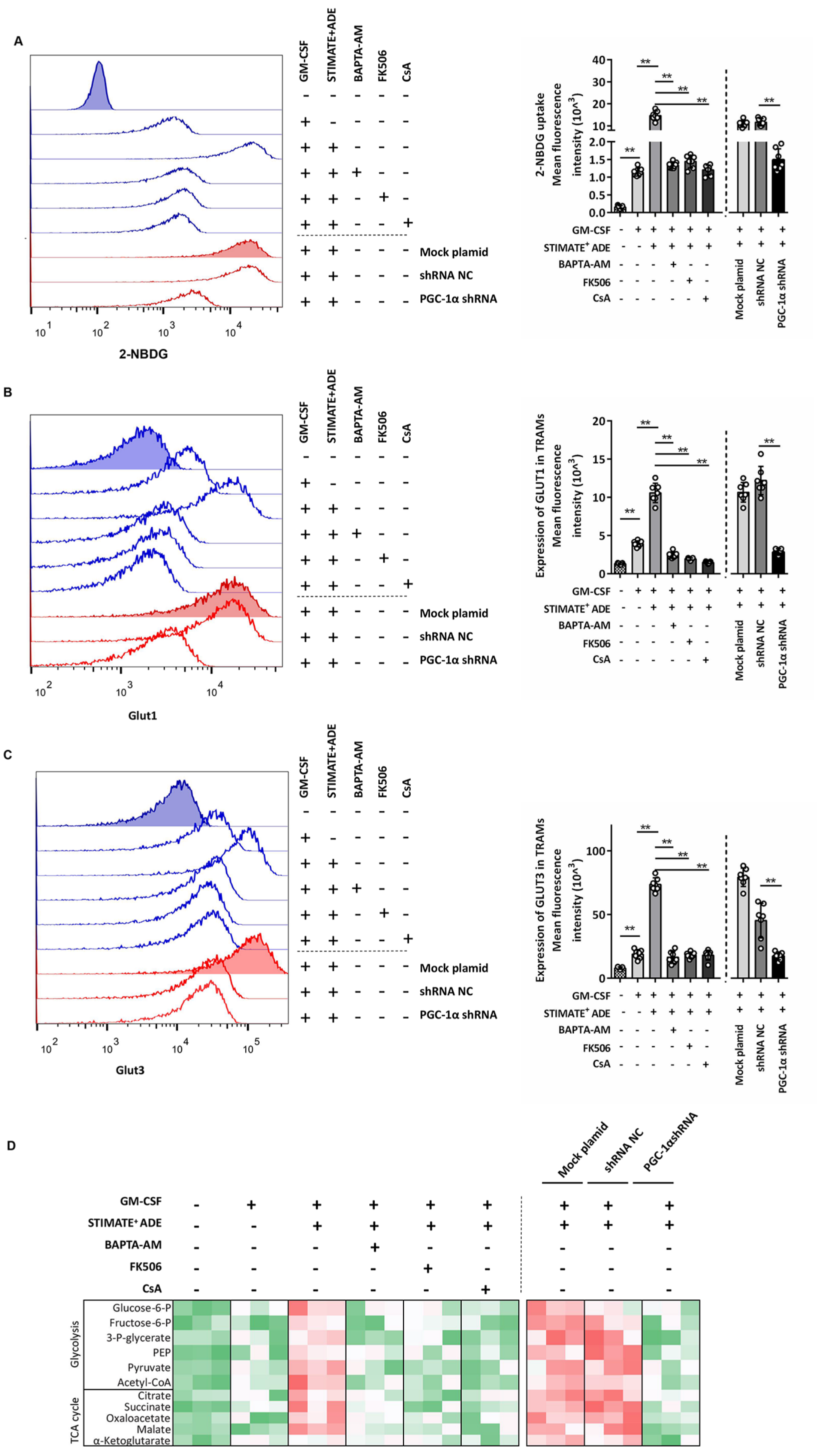

Figure S12. Blockade of Ca^{2+}-CaN-PGC-1α signaling affects glucose utilization of TRAMs

(A) Flow cytometry analysis of the fluorescence intensity of glucose mimetic 2-NBDG uptake in WT or STIMATE$^{-/-}$ mouse TRAMs induced by GM-CSF after cocultivation with STIMATE^{+}ADE, BAPTA-AM (1.5 μM), FK506 (100 μg/L), CsA (7 nM) and PGC-1α shRNA (2 μg).

(B-C) Flow cytometry analysis of the fluorescence intensity of the glucose transporters GLUT1 and GLUT3 in WT or STIMATE$^{-/-}$ mouse TRAMs induced by GM-CSF after cocultivation with STIMATE^{+}ADE, BAPTA-AM (1.5 μM), FK506 (100 μg/L), CsA (7 nM) and PGC-1α shRNA (2 μg).

(D) The abundance of polar metabolites of the tricarboxylic acid cycle and the glycolytic pathway in WT or STIMATE$^{-/-}$ mouse TRAMs induced by GM-CSF after cocultivation with STIMATE^{+}ADE, BAPTA-AM (1.5 μM), FK506 (100 μg/L), CsA (7 nM) and PGC-1α shRNA (2 μg). Heatmap showing log normalized values from minimum (blue) to maximum (red).

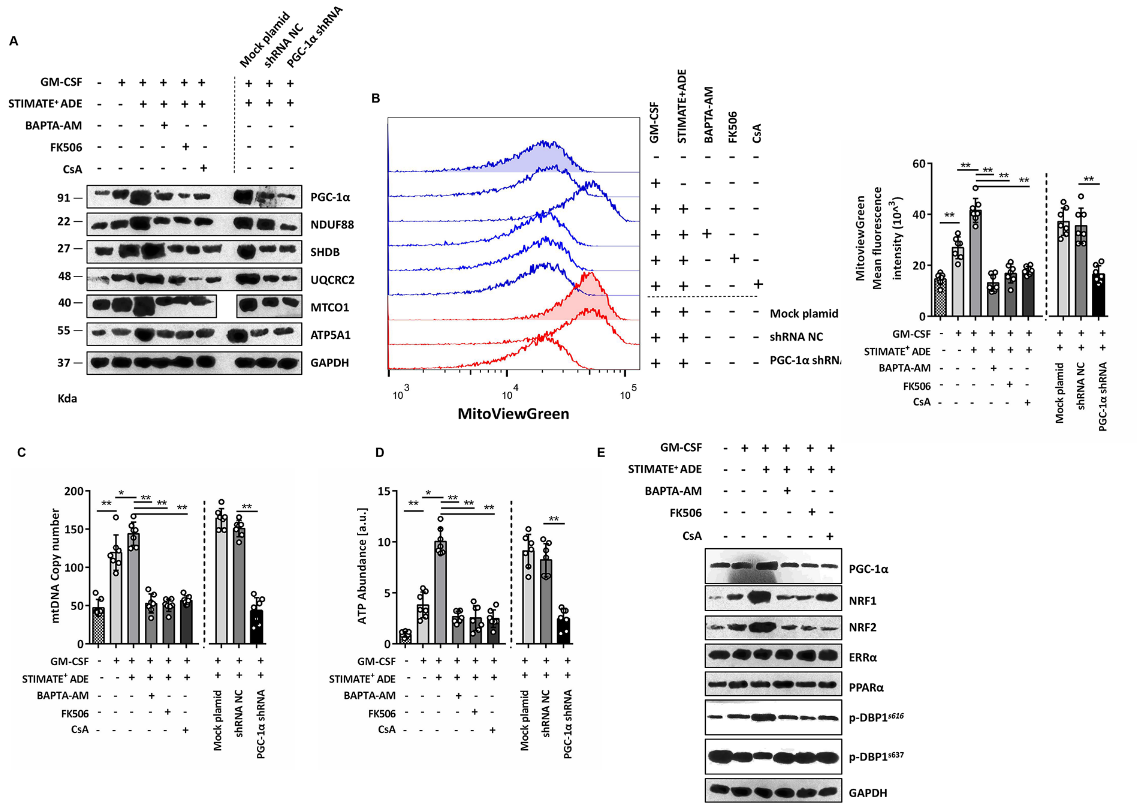

Figure S13. Blockade of Ca^{2+}-CaN-PGC-1α signaling affects mitochondrial biosynthesis of TRAMs.

(A) Representative Western blotting image of mtDNA-coding ETC protein expression (NDUF88, SHDB, UQCRC2, MTCO1, ATP5A1) in WT or STIMATE$^{-/-}$ mouse TRAMs induced by GM-CSF after cocultivation with STIMATE^{+}ADE, BAPTA-AM (1.5 μM), FK506 (100 μg/L), CsA (7 nM) and PGC-1α shRNA (2 μg). n=3.

(B) Flow cytometry analysis of the mitochondrial volume by MitoViewGreen Fluorescence staining in WT or STIMATE$^{-/-}$ mouse TRAMs induced by GM-CSF after cocultivation with STIMATE^{+}ADE, BAPTA-AM (1.5 μM), FK506 (100 μg/L), CsA (7 nM) and PGC-1α shRNA (2 μg). The right figure shows the MFI (495 nm absorption wavelength/516 nm emission wavelength) quantification of MitoViewGreen in TRAMs.

(C-D) mtDNA copy number and ATP abundance by qPCR and LC–MS in WT or STIMATE$^{-/-}$ mouse TRAMs induced by GM-CSF after cocultivation with STIMATE^{+}ADE, BAPTA-AM (1.5 μM), FK506 (100 μg/L), CsA (7 nM) and PGC-1α shRNA (2 μg).

(E) Representative Western blotting image of NTFs (PPARα and ERRα) and TFms (NRF1 and NRF1) and phosphorylating DPR1 in WT or STIMATE$^{-/-}$ mouse TRAMs induced by GM-CSF after cocultivation with STIMATE^{+}ADE, BAPTA-AM (1.5 μM), FK506 (100 μg/L), CsA (7 nM) and PGC-1α shRNA (2 μg).

Data are shown as the mean±SD, *p < 0.05, and **p < 0.01 by analysis of variance (ANOVA) followed by a post hoc test compared to resting TRAMs. All data were derived from 3 independent experiments.

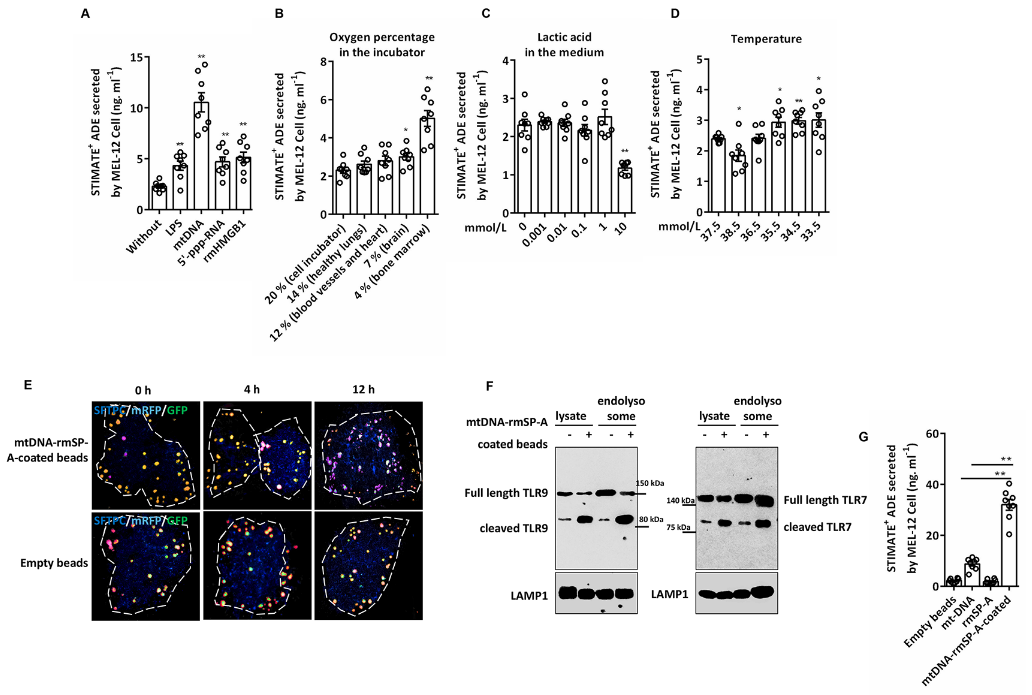

Figure S14. Production of therapeutic STIMATE⁺ADE.

(A) Exo-based ELISA to detect the concentration of STIMATE⁺ADE released by MEL-12 cells stimulated with LPS and rmHMGB1 or transfected with mtDNA and 5'-pppRNA.

(B) ELISA to detect the concentration of STIMATE⁺ADE released by MEL-12 cells at different oxygen levels, referring to the oxygen content of human tissue.

(C) The concentration of STIMATE⁺ADE released by MEL-12 cells in lactic acid-supplemented medium at the indicated concentrations.

(D) The concentration of STIMATE⁺ADE released by MEL-12 cells at the specified ambient temperature.

(E) Fluorescence images of mtDNA-rmSP-A-coated beads at 4 h and 12 h after being ingested by MLE-12. The basic mCherry-GFP beads show yellow fluorescence outside the cell or in endosomes. When endolysosomes are formed, acid-sensitive GFP fluorescence is quenched, while mCherry is unaffected, resulting in red fluorescence of the beads.

(F) Lysates or phagosome preparations from ingested mtDNA-rmSP-A-coated beads 4 h and 12 h MLE-12 cells fed latex beads were separated by SDS–PAGE and probed with antibodies specific for the indicated proteins. Full-length TLR9/TLR7 and the truncated protein (cleaved TLR9/TLR7) are indicated.

(G) exosomes-based ELISA detected the levels of STIMATE⁺-ADE released by MLE12 cells induced by mtDNA-rmSP-A-coated beads. Empty bait and mtDNA transfection served as a control group.

Data are shown as the mean±SD, *p < 0.05, and **p < 0.01 by analysis of variance (ANOVA) followed by a post hoc test compared to resting TRAMs. All data were derived from 3 independent experiments.

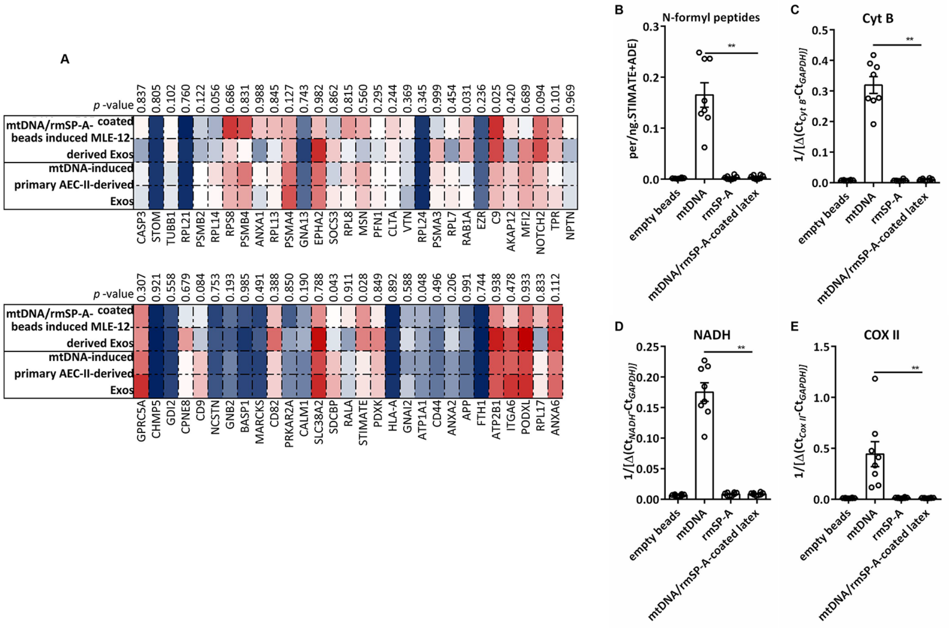

Figure S15. Quality Control of mtDNA/rmSP-A-coated beads induced MLE-12-derived STIMATE⁺ADE

(A) Comparison of overlapping differential protein cargoes in mtDNA/rmSP-A-coated beads induced MLE-12-derived STIMATE⁺ADE and mtDNA transfection induced primary AEC-IIs-derived STIMATE⁺ADE.

(B-D) The levels of free NFP and mtDNA Cyt B, NADH and COX II in therapeutic mtDNA/rmSP-A-coated beads induced by MLE-12-derived STIMATE⁺ADE were detected by ELISA and qPCR. Data are shown as the mean±SD, *p < 0.05, and **p < 0.01 by analysis of variance (ANOVA) followed by a post hoc test compared to resting TRAMs. All data were derived from 3 independent experiments.

Supplementary Table.

Table 1. Characteristics of the ALI/ARDS patients

	ALI/ARDS
n	**112**
Age (yr)	51.5 ± 7.3
Male (%)	47.3
APACHE III	69.1 ± 20.2
Infection (%)	72(64.3%)
Sepsis (%)	47(42%)
Trauma (%)	40(35.7%)
Shock (%)	27(24.1%)

Values are means ± SD

APACHE III: Acute physiology and chronic health evaluation III

Table 2. Characteristics of the IFP patients

	PF
n	44
Age (yr)	68.5 ± 7.2
Male (%)	88.6
Smorking (%)	72.7
IPF (%)	6.8
SSc–ILD (%)	18.2
RA–ILD (%)	6.8
Sarcoidosis, fibrotic (stage IV) (%)	34.1
Chronic fibrotic hypersensitivity pneumonitis (%)	2.3
Unclassifiable fibrotic ILD (%)	31.8
FVC (% of predicted value)	66.8 ± 8.2
FEV_1:FVC	0.83 ± 0.02
Carbon monoxide diffusing capacity (% of predicted value)	41.7 ± 9.3
Distance of 6 min test (m)	385 ± 72.6
Use of Supplemental oxygen (%)	27.3
Time since diagmosis (yr)	2.4 ± 1.7

Values are means ± SD

IPF: idiopathic pulmonary fibrosis

SSc: systemic sclerosis

RA: Rheumatoid arthritis

ILD: interstitial lung disease

FEV1/FVC: Denotes forced expiratory volume in one second

FVC: Forced vital capacity

Denotes forced expiratory volume in one second

Table 3.Information of antibody.

Product #	Name	Species Reactivity	Host	Conjugate	Manufacturer
MHCD4522	CD45	Human	Mouse/IgG1	PE-Alexa Fluor 610	Invitrogen
15-0118-42	CD11B	Human	Mouse/IgG1, kappa	PE-Cyanine5	eBioscience
78-0116-42	CD11C	Human	Mouse/IgG1, kappa	Super Bright 780	eBioscience
48-0649-42	CD64	Human	Mouse/IgG1, kappa	eFluor 450	eBioscience
61-0451-82	CD45	Mouse	Rat/IgG2b, kappa	PE-Alexa Fluor 610	eBioscience
15-0112-82	CD11B	Mouse	Rat/IgG2b, kappa	PE-Cyanine5	eBioscience

78-0114-82	CD11C	Mouse	Armenian hamster	Super Bright 780	eBioscience
12-0641-82	CD64	Mouse	Mouse/IgG1, kappa	PE	eBioscience
A19635	IL-1β	Mouse	Rabbit	UnConjugate	abclonal
A3774	iNOS	Mouse	Rabbit	UnConjugate	abclonal
A12255	IL-10	Mouse	Rabbit	UnConjugate	abclonal
A0694	SOCS3	Mouse	Rabbit	UnConjugate	abclonal
A18692	TGF-β	Mouse	Rabbit	UnConjugate	abclonal
PA5-44449&PA5-70169	STIMATE	Human, Mouse	Rabbit	UnConjugate	Invitrogen
A5789	CD63	Mouse	Rabbit	UnConjugate	abclonal
A4326	TSG101	Mouse	Rabbit	UnConjugate	abclonal
A2582	ERP72	Mouse	Rabbit	UnConjugate	abclonal
AC038	LAMP1	Mouse	Rabbit	UnConjugate	abclonal
A15788	Sec61β	Human, Mouse	Rabbit	UnConjugate	abclonal
A9764	STIM1	Mouse	Rabbit	UnConjugate	abclonal
A19278	Na+K+ATPase	Mouse	Rabbit	UnConjugate	abclonal
A7412	ORAI1	Mouse	Rabbit	UnConjugate	abclonal
A1934	Rab27a	Human & Mouse	Rabbit	UnConjugate	abclonal
A2094	Ki67	Human & Mouse	Rabbit	UnConjugate	abclonal
A11727	GLUT1	Human & Mouse	Rabbit	UnConjugate	abclonal
A4137	GLUT3	Human & Mouse	Rabbit	UnConjugate	abclonal
A12348	PGC-1α	Human & Mouse	Rabbit	UnConjugate	abclonal
A3252	NRF-1	Human & Mouse	Rabbit	UnConjugate	abclonal

A4176	ERRα	Human & Mouse	Rabbit	UnConjugate	abclonal
A6697	PPARα	Human & Mouse	Rabbit	UnConjugate	abclonal
ab110413	Total OXPHOS Rodent WB Cocktail	Human & Mouse	Rabbit	UnConjugate	abcam
A19065	GAPDH	Human & Mouse	Rabbit	UnConjugate	abclonal
Cat# A-21245	Alexa647-conjugated anti-rabbit-IgG			Alexa647	Molecular Probes
Cat# A-21422	Alexa555-conjugated anti-mouse-IgG			Alexa555	Molecular Probes
AE012	Mouse anti GFP-Tag mAb			UnConjugate	abclonal
#4283	MyD88	Mouse	Rabbit	UnConjugate	Cell Signaling Technology
#74792	Mitochondrial Dynamics Antibody Sampler Kit II	Mouse	Rabbit	UnConjugate	Cell Signaling Technology

Supplementary Table 4. Sequence ofoligonucleotides

**sgRNA oligonucleotides:**	
mouse STIMATE guide 1:	3'-GAGGGCGCACTGTCCTACCC-5'
mouse STIMATE guide 2:	3'-GGTACGTACCATATCCTCCA-5'
mouse RAB27A guide 1:	3'-CCAAGGCCAAGAACTTGATG-5'
mouse RAB27A guide 2:	3'-CACAGTGGGCATTGATTTCA-5'
**shRNA oligonucleotides:**	
mouse PGC-1α shRNA-1	3'-GCTCCATCCTTTGGCCATTATC-5'
mouse PGC-1α shRNA-2	3'-GCAGATGCCGGGAAAGAAAGA-5'
mouse PGC-1α shRNA-NC-1	3'-GCCTCTTCCACTGTGTATACCT-5'
mouse PGC-1α shRNA-NC-2	3'-GACGAGGCCTAGAGAGAGAAA-5'
**Primer oligonucleotides**	

mouse PGC-1α	Forward: GGAGGCGAGAACATCAAGCC
	Reverse: CGGCCTTCCCTCGTAGTGA
mouse GAPDH	Forward: TTGATGGCAACAATCTCCAC
	Reverse: CGTCCCGTAGACAAAATGGT
**mtDNA Primer oligonucleotides**	
mouse mtDNA region 1	Forward: TGAACGGCTAAACGAGGGTC
	Reverse: AGCTCCATAGGGTCTTCTCGT
mouse mtDNA region 2	Forward: CAGTCCCCTCCCTAGGACTT
	Reverse: ACCCTGGTCGGTTTGATGTT
mouse mtDNA region 3	Forward: TAATCGCACATGGCCTCACA
	Reverse: GAAGTCCTCGGGCCATGATT
mouse gDNA B2m	Forward: AGCAAAGAGGCCTAATTGAAGTC
	Reverse: GAAGTAGCCACAGGGTTGGG
mouse gDNA Tuba1a	Forward: TGAGGAGGTTGGTGTGGATTC
	Reverse: TGAGGAGGTTGGTGTGGATTC

Supplementary Table 5. The overlap of differences proteomics in exosome induced by DAPM & PAMP

GPRC5A	G protein-coupled receptor class C group 5 member A
CHMP5	Charged multivesicular body protein 5
GDI2	Rab GDP dissociation inhibitor beta
CPNE8	Copine-8
CD9	CD9 antigen
NCSTN	Nicastrin
GNB2	Guanine nucleotide-binding protein G(I)/G(S)/G(T) subunit beta-2
BASP1	Brain acid soluble protein 1
MARCKS	Myristoylated alanine-rich C-kinase substrate
CD82	CD82 antigen
PRKAR2A	cAMP-dependent protein kinase type II-alpha regulatory subunit
CALM1	Calmodulin 1
SLC38A2	Sodium-coupled neutral amino acid transporter 2
SDCBP	Syndecan binding protein
RALA	Ras-related protein Ral-A
STIMATE	STIM activating enhancer
PDXK	Pyridoxal kinase
HLA-A	HLA class I histocompatibility antigen, A-33 alpha chain
GNAI2	Guanine nucleotide-binding protein G(i) subunit alpha-2
ATP1A1	ATPase Na+/K+ transporting subunit alpha 1
CD44	CD44 antigen
ANXA2	Isoform 2 of Annexin A2
APP	Amyloid beta A4 protein
FTH1	Ferritin heavy chain
ATP2B1	Plasma membrane calcium-transporting ATPase 1

ITGA6	Integrin alpha-6
PODXL	Podocalyxin
RPL17	Isoform 3 of 60S ribosomal protein L17
ANXA6	Annexin A6
CASP3	Caspase 3
STOM	Erythrocyte band 7 integral membrane protein
TUBB1	Tubulin beta-1 chain
RPL21	60S ribosomal protein L21
PSMB2	Proteasome subunit beta type-2
RPL14	60S ribosomal protein L14
RPS8	40S ribosomal protein S8
PSMB4	Proteasome subunit beta type-4
ANXA1	Annexin A1
RPL13	60S ribosomal protein L13
PSMA4	Proteasome subunit alpha type-4;
GNA13	Guanine nucleotide-binding protein subunit alpha-13
EPHA2	Ephrin type-A receptor 2
SOCS3	Suppressor of cytokine signaling 3
RPL8	60S ribosomal protein L8
MSN	Moesin
PFN1	Profilin-1
CLTA	Clathrin light chain A
VTN	Vitronectin
RPL24	60S ribosomal protein L24
PSMA3	Proteasome subunit alpha type-3
RPL7	60S ribosomal protein L7
RAB1A	Ras-related protein Rab-1A
EZR	Ezrin
C9	Complement component C9
AKAP12	A-kinase anchor protein 12
MFI2	Melanotransferrin
NOTCH2	Neurogenic locus notch homolog protein 2
TPR	Nucleoprotein TPR
NPTN	Neuroplastin
PPP2CA	Serine/threonine-protein phosphatase 2A catalytic subunit alpha isoform
RPS4Y1	40S ribosomal protein S4, Y isoform 1
ITGB4	Integrin beta-4
NCL	Nucleolin
HNRNPK	Isoform 2 of Heterogeneous nuclear ribonucleoprotein K
CLSTN1	Calsyntenin-1
EEF2	Elongation factor 2
TUBB4B	Tubulin beta-4B chain
TRIM28	Transcription intermediary factor 1-beta

HNRNPA3	Heterogeneous nuclear ribonucleoprotein A3
HSP90AB1	Heat shock protein HSP 90-beta
PRMT1	Protein arginine N-methyltransferase 1
ACTB	Actin, cytoplasmic 1
GNB2L1	Guanine nucleotide-binding protein subunit beta-2-like 1
ARPC2	Actin-related protein 2/3 complex subunit 2
KIF5B	Kinesin family member 5B
PRPF19	Pre-mRNA-processing factor 19
HNRNPA1	Heterogeneous nuclear ribonucleoprotein A1
SEPT2	Isoform 2 of Septin-2
THBS1	Thrombospondin-1
LAMA5	Laminin subunit alpha-5
FBN1	Fibrillin-1
SPOCK1	SPARC (osteonectin), cwcv and kazal like domains proteoglycan 1
